# The genetic ancestry of American Creole cattle inferred from uniparental and autosomal genetic markers

**DOI:** 10.1038/s41598-019-47636-0

**Published:** 2019-08-07

**Authors:** Catarina Ginja, Luis Telo Gama, Oscar Cortés, Inmaculada Martin Burriel, Jose Luis Vega-Pla, Cecilia Penedo, Phil Sponenberg, Javier Cañón, Arianne Sanz, Andrea Alves do Egito, Luz Angela Alvarez, Guillermo Giovambattista, Saif Agha, Andrés Rogberg-Muñoz, Maria Aparecida Cassiano Lara, BioBovis Consortium, Sónia Afonso, Lenin Aguirre, Eileen Armstrong, Maria Esperanza Camacho Vallejo, Amado Canales, Bernardo Cassamá, Gloria Contreras, J. M. Moras Cordeiro, Susana Dunner, Ahmed Elbeltagy, Maria Clorinda Soares Fioravanti, Mayra Gómez Carpio, Mariano Gómez, Antonio Hernández, Darwin Hernandez, Raquel Soares Juliano, Vincenzo Landi, Ribamar Marques, Rubén D. Martínez, O. Roberto Martínez, Lilia Melucci, Baldomero Molina Flores, Fernando Mújica, Pere-Miquel Parés i Casanova, Jorge Quiroz, Clementina Rodellar, Gerald Tjon, Tumininu Adebambo, Odalys Uffo, Julio César Vargas, Axel Villalobos, Pilar Zaragoza, Juan Vicente Delgado, Amparo Martinez

**Affiliations:** 1 CIBIO/InBIO, Centro de Investigação em Biodiversidade e Recursos Genéticos, Universidade do Porto, Porto, Portugal; 2 CIISA.Faculdade de Medicina Veterinaria, Universidade de Lisboa, Lisbon, Portugal; 3 Departamento de Producción Animal, Facultad de Veterinaria, Universidad Complutense de Madrid, Madrid, Spain; 4 Laboratorio de Genética Bioquímica, Facultad de Veterinaria, Universidad de Zaragoza, Zaragoza, Spain; 5 Laboratorio de Investigación Aplicada, Servicio de Cría Caballar de las Fuerzas Armadas, Córdoba, Spain; 6 Veterinary Genetics Laboratory, University of California, Davis, California USA; 7 Virginia-Maryland Regional College of Veterinary Medicine. Virginia Tech, Virginia, USA; 8 Embrapa Gado de Corte, Campo Grande, Brazil; 9 Universidad Nacional de Colombia, Sede Palmira, Colombia; 10 Facultad de Ciencias Veterinarias, Universidad Nacional de La Plata, La Plata, Argentina; 11 Animal Production Department, Faculty of Agriculture, Ain Shams University, Cairo, Egypt; 12 CONICET, Buenos Aires, Argentina; 13 Instituto de Zootecnia, Centro de Genética e Reprodução, Nova Odessa-SP, Brazil; 14 Departamento de Genética, Facultad de Veterinaria, Universidad de Córdoba, Córdoba, Spain; 15 Animal Beeding Consulting S.L. Universidad de Córdoba, Córdoba, Spain; 16 Faculdade de Veterinária, Universidade Eduardo Mondlane, Maputo, Mozambique; 17 Universidad Nacional de Loja, Loja, Ecuador; 18 Departamento de Genética y Mejora Animal, Facultad de Veterinaria-UdelaR, Montevideo, Uruguay; 19 IFAPA centro Alameda del Obispo, Córdoba, Spain; 20 Direçao Geral da Pecuária, Bissau, Guinea-Bissau; 21 Instituto Nacional de Investigaciones Agrícolas (INIA)-Venezuela, Maracay, Venezuela; 22 Faculdade de Medicina Veterinária, Universidade José Eduardo dos Santos, Huambo, Angola; 23 Department of Animal Biotechnology, Animal Production Research Institute, Ministry of Agriculture, Cairo, Egypt; 24 Universidade Federal de Goiás, Goiânia, Goiás Brazil; 25 Servicio de Ganadería, Diputación Foral de Bizkaia, Bizkaia, Spain; 26 Universidad Veracruzana, Veracruz, Mexico; 27 Embrapa Pantanal, Corumbá-MS, Brazil; 28 EMBRAPA Amazônia Oriental, Belém, Pará Brazil; 29 Facultad de Ciencias Agrarias, Universidad Nacional de Lomas de Zamora, Zamora, Argentina; 30 Universidad Nacional de Asunción, Asunción, Paraguay; 31 Facultad de Ciencias Agrarias, Universidad Nacional de Mar del Plata, Balcarce, Argentina; 32 Facultad de Ciencias Agrarias, Universidad Austral de Chile, Santiago, Chile; 33 Universitat de Lleida, Lleida, Spain; 34 Instituto Nacional de Investigaciones Forestales, Agrícolas y Pecuarias, Mexico; 35 Ministry of Agriculture, Animal Husbandry and Fisheries, Paramaribo, Suriname; 36 University of Agriculture Abeokuta, Abeokuta, Nigeria; 37 Centro Nacional de Sanidad Agropecuaria, La Habana, Cuba; 38 Universidad Estatal Amazónica, Puyo, Pastaza Ecuador; 39 Instituto de Investigación Agropecuaria. Estación Experimental El Ejido, Los Santos, Panama

**Keywords:** Animal breeding, Structural variation

## Abstract

Cattle imported from the Iberian Peninsula spread throughout America in the early years of discovery and colonization to originate Creole breeds, which adapted to a wide diversity of environments and later received influences from other origins, including zebu cattle in more recent years. We analyzed uniparental genetic markers and autosomal microsatellites in DNA samples from 114 cattle breeds distributed worldwide, including 40 Creole breeds representing the whole American continent, and samples from the Iberian Peninsula, British islands, Continental Europe, Africa and American zebu. We show that Creole breeds differ considerably from each other, and most have their own identity or group with others from neighboring regions. Results with mtDNA indicate that T1c-lineages are rare in Iberia but common in Africa and are well represented in Creoles from Brazil and Colombia, lending support to a direct African influence on Creoles. This is reinforced by the sharing of a unique Y-haplotype between cattle from Mozambique and Creoles from Argentina. Autosomal microsatellites indicate that Creoles occupy an intermediate position between African and European breeds, and some Creoles show a clear Iberian signature. Our results confirm the mixed ancestry of American Creole cattle and the role that African cattle have played in their development.

## Introduction

Cattle did not exist in the Americas until the end of the 15th century, when Columbus arrived with various livestock species on his second journey to the New World^1^. Since then, cattle from various regions have been brought to the New World, but the origins and major genetic influences received by American Creole cattle have been controversial and the subject of intense debate. Broadly, it is believed that an initial foundation stock was developed from a narrow base established from cattle brought from Portugal and Spain with the first settlements, but these quickly multiplied and by the end of the 16th century cattle and other livestock had expanded throughout the continent in large numbers^2,3^. As Creole cattle breeds went through this initial period of rapid expansion throughout the American continent they played a crucial role as a source of labor, food and hides, and since then have evolved to adapt to an extremely diverse set of environmental conditions which cover climates as varied as the Great Plains of North America, the semiarid area in Northeast Brazil, the tropical regions of the Caribbean or the mountains and glaciers of Patagonia. These cattle therefore represent an extremely valuable biological resource to understand the genetic background that may be implicated in various mechanisms of adaptation, including adjustment to climate change, resistance to parasites, ability to utilize different feedstuffs, etc.^4^.

The dispersion of cattle throughout the Americas essentially followed the colonization paths established by Portugal and Spain. The Spanish colonization route used the Antilles as first base, and it is often assumed that dissemination of livestock followed three major routes: (1) from Cuba to Mexico and later on to North America; (2) from the Caribbean to Venezuela and Colombia; (3) through Rio de la Plata to Peru, Bolivia, Paraguay, Chile, Argentina and Uruguay^1^. The Portuguese path disseminated livestock through the colonial captaincies in the coast of Brazil, from animals received both from mainland Portugal and from the of Cape Verde Islands^5^.

Initially, the voyage from the Iberian Peninsula to America usually had a stopover in Cape Verde or in the Canary Islands, where ships obtained supplies, including animals, for the remaining stretch of the journey^5^. It has been argued that animals from Africa could have influenced the development of Creole cattle^2,5,6^ because these islands are in close proximity to Africa, or under the assumption that livestock may have accompanied slave trade routes. Current evidence tends to support this hypothesis in cattle from the Caribbean^7,8^ and other parts of the Americas^9,10^. Nevertheless, it has not been possible to disentangle whether the likely influence of African cattle on American Creoles has been achieved directly through animals arriving from Africa or indirectly through Iberian cattle, as an African signature has also been revealed in Iberian breeds^10–12^. Starting in the 17th century, European cattle were brought in large numbers to the Americas, and from the beginning of the 20th century *Bos indicus* from India were extensively crossed with local populations, particularly in tropical areas of the Americas^3^. Thus, bulls imported from India were backcrossed with local Creole cattle, to originate the various American indicine breeds currently recognized.

New challenges in the livestock sector include indiscriminate crossbreeding and replacement by more productive transboundary breeds which has led to the decline of Creole populations as they have been progressively abandoned or were admixed with exotic germplasm. This trend has resulted in the loss or near-extinction of the majority of Creole cattle populations, in spite of their uniqueness and long-term adaptation to various environmental conditions. Nonetheless, conservation programs have been established for some of the Creole populations in order to preserve an important resource in terms of overall genetic diversity, and for the key role they play in social economy and community cultural identity in Latin America^3,13,14^.

Over the last few years, various studies have been accomplished with the goal of investigating the origins and genetic structure of Creole cattle. These studies, however, have been generally limited to a specific type of genetic marker such as uniparental markers^8,10,11,15^ and microsatellites^16^ or have used a narrow sample of the extant Creole breeds and their Iberian ancestors^17–19^.

In this study, we analyzed DNA samples obtained from 114 cattle breeds distributed worldwide, including a comprehensive representation of 40 Creole cattle breeds covering the whole American continent. In addition, we also analyzed cattle DNA samples from the Iberian Peninsula, British islands, Continental Europe, Africa and American zebu, as potential sources of genetic influence on Creole cattle. For this study, we have expanded significantly our sampling of Creole populations analyzed in previous studies and the breeds that may have influenced them, including novel information on African cattle breeds.

For this work, a large consortium of Ibero-American researchers used the geoevolutionary significance of animal genetic resources in the Americas and their routes of dissemination to study their origins, considering the impact that these breeds have on culture and traditions, and the fact that they have been developed over centuries of adaptation. We have studied the genetic diversity and relationships of these breeds to establish the foundations and to design a continent-wide program for the characterization, conservation, recognition and valuation of the zoogenetic heritage of this region. These results are of crucial importance to the sustainable development and creation of wealth, especially for the underdeveloped regions and human communities which have acted as guardians of these farm animal genetic resources over the last century^20^.

We used uniparental and autosomal genetic markers to investigate the patterns of colonization and expansion of the Creole genetic pool, using various statistical tools for more meaningful analyses of their phylogeographic history and population structure. In particular, mitochondrial DNA sequence data and Y-chromosome haplotype information were increased by more than half of that reported in our previous studies, but also for autosomal microsatellite markers with novel information on 29 breeds and 1,114 individuals newly genotyped from various regions.

We combined the information revealed by the various genetic markers to investigate: (1) the diversity, identity and genetic structure of Creole cattle, as disclosed by the different sources of information; (2) the evidence of a direct African influence in the development of Creoles; (3) the signals of an Iberian genetic signature still remaining in current Creole populations, (4) the extent of *Bos indicus* introgression in the gene pool of Creole cattle populations.

## Results

The information on mitochondrial DNA sequence data, Y-chromosome haplotype and autosomal microsatellite markers included in our study is shown in Table 1. We expanded significantly our previous sampling of Creole cattle and the breeds that influenced them, including African cattle. Overall, we studied a total of 4,658 animals from 114 cattle breeds, including 1,480 Creole from 40 breeds, 1,930 Iberian from 39 breeds, 556 African from 18 breeds, 271 British from 6 breeds, 229 Continental European from 6 breeds, and 192 Indicine from 5 breeds sampled in the Americas.

**Table 1:** Information on the cattle breeds and geographic groups included in the analysis of mitochondrial, Y-chromosome and autosomal microsatellite markers.

Breed group	Country of origin	Breed name	Breed Code	Acronym	Microstellites		mtDNA		Ychr	
					N	Reference	N	Reference	N	Reference
Creole	Argentina	Criollo Argentino	34	AM_ARG	50	Martínez *et al*.^16^	23	Ginja *et al*.^10^	18	Ginja *et al*.^10^
Creole	Argentina	Criollo Patagónico	35	AM_PAT	35	Martínez *et al*.^16^	10	This study	8	This study
Creole	Bolivia	Criollo Yacumeño	30	AM_YAC	32	This study	19	This study	29	This study
Creole	Brazil	Caracú	25	AM_CAR	74	Martínez *et al*.^16^	10	Ginja *et al*.^10^	73	Ginja *et al*.^10^
Creole	Brazil	Crioulo Lagueano	26	AM_LAG	39	Egito *et al*.^28^	11	This study	25	This study
Creole	Brazil	Curraleiro	27	AM_CUR	50	Egito *et al*.^28^	10	This study	25	This study
Creole	Brazil	Mocho Nacional	28	AM_MNA	50	Egito *et al*.^28^	7	This study	20	This study
Creole	Brazil	Pantaneiro	29	AM_PAN	48	Egito *et al*.^28^	9	This study	25	This study
Creole	Chile	Criollo Patagónico Chileno	36	AM_PCH	38	This study	16	This study	38	This study
Creole	Colombia	Blanco Orejinegro	13	AM_BON	25	Martínez *et al*.^16^	14	This study	8	This study
Creole	Colombia	Caqueteño	14	AM_CAQ	25	Martínez *et al*.^16^	12	This study	5	This study
Creole	Colombia	Casanareño		AM_CAS					6	This study
Creole	Colombia	Chino Santandereano	18	AM_CHS	25	Martínez *et al*.^16^	25	This study	5	This study
Creole	Colombia	Costeño con Cuernos	17	AM_CCC	25	Martínez *et al*.^16^	11	This study	3	This study
Creole	Colombia	Hartón del Valle	21	AM_HVA	22	Martínez *et al*.^16^	12	This study	11	This study
Creole	Colombia	Lucerna	20	AM_LUC	23	Martínez *et al*.^16^	11	This study		
Creole	Colombia	Romosinuano	16	AM_RMS	25	Martínez *et al*.^16^	15	This study	10	This study
Creole	Colombia	Sanmartinero	15	AM_SMA	24	Martínez *et al*.^16^	10	This study	4	This study
Creole	Colombia	Velasquez	19	AM_VEL	25	Martínez *et al*.^16^	15	This study	4	This study
Creole	Cuba	Criollo Cubano	38	AM_CUB	50	Martínez *et al*.^16^	7	This study	16	This study
Creole	Cuba	Siboney	39	AM_SIB	50	Martínez *et al*.^16^				
Creole	Ecuador	Criollo Ecuatoriano	23	AM_ECU	46	Martínez *et al*.^16^			3	This study
Creole	Ecuador	Criollo Macabeo	24	AM_MAC	25	Vargas *et al*. 2016				
Creole	Mexico	Criollo Baja California	6	AM_CBC	20	Martínez *et al*.^16^	20	Ginja *et al*.^10^		
Creole	Mexico	Criollo Chiapas	9	AM_CHI	30	Martínez *et al*.^16^	15	Ginja *et al*.^10^	12	Ginja *et al*.^10^
Creole	Mexico	Criollo Chihuahua	7	AM_CHU	16	Martínez *et al*.^16^	19	Ginja *et al*.^10^	4	Ginja *et al*.^10^
Creole	Mexico	Criollo Lechero Tropical	4	AM_CRI	46	This study				
Creole	Mexico	Criollo Nayarit	8	AM_CNY	24	Martínez *et al*.^16^	16	Ginja *et al*.^10^		
Creole	Mexico	Criollo Poblano	5	AM_POB	42	Martínez *et al*.^16^	16	This study		
Creole	Panama	Guabalá	10	AM_GUA	25	Martínez *et al*.^16^	10	Ginja *et al*.^10^	14	This study
Creole	Panama	Guaymí	11	AM_GUY	36	Martínez *et al*.^16^	15	This study	8	This study
Creole	Paraguay	Criollo Pilcomayo	33	AM_PIL	36	Martínez *et al*.^16^				
Creole	Paraguay	Pampa Chaqueño	32	AM_PAC	50	Martínez *et al*.^16^	16	Ginja *et al*.^10^	25	Ginja *et al*.^10^
Creole	Saint Croix Island (Caribe)	Senepol	37	AM_SEN	22	This study	14	This study	9	This study
Creole	Suriname	Suriname	12	AM_SUR	50	This study				
Creole	Uruguay	Criollo Uruguayo	31	AM_CRU	43	Martínez *et al*.^16^	11	This study	25	This study
Creole	USA	Florida Cracker	2	AM_FCR	50	This study	13	This study	6	This study
Creole	USA	Pineywoods	3	AM_PIW	50	This study	18	This study	9	This study
Creole	USA	Texas Longhorn	1	AM_TLH	80	Martínez *et al*.^16^	16	Ginja *et al*.^10^	49	Ginja *et al*.^10^
Creole	Venezuela	Criollo Limonero	22	AM_LIM	48	Martínez *et al*.^16^	14	This study	23	This study
Iberian	Portugal	Alentejana	66	PT_ALT	38	Martínez *et al*.^16^	16	Ginja *et al*.^10^	31	Ginja *et al*.^10^
Iberian	Portugal	Arouquesa	67	PT_ARO	70	Martínez *et al*.^16^	16	Ginja *et al*.^10^	31	Ginja *et al*.^10^
Iberian	Portugal	Barrosã	68	PT_BAR	69	Martínez *et al*.^16^	16	Ginja *et al*.^10^	33	Ginja *et al*.^10^
Iberian	Portugal	Brava de Lide	69	PT_BRA	43	Martínez *et al*.^16^	16	Ginja *et al*.^10^	26	Ginja *et al*.^10^
Iberian	Portugal	Cachena	70	PT_CAC	51	Martínez *et al*.^16^	16	Ginja *et al*.^10^	25	Ginja *et al*.^10^
Iberian	Portugal	Garvonesa	71	PT_GAR	39	Martínez *et al*.^16^	16	Ginja *et al*.^10^	6	Ginja *et al*.^10^
Iberian	Portugal	Marinhoa	72	PT_MRI	46	Martínez *et al*.^16^	16	Ginja *et al*.^10^	17	Ginja *et al*.^10^
Iberian	Portugal	Maronesa	73	PT_MAR	47	Martínez *et al*.^16^	16	Ginja *et al*.^10^	23	Ginja *et al*.^10^
Iberian	Portugal	Mertolenga	74	PT_MER	64	Martínez *et al*.^16^	16	Ginja *et al*.^10^	17	Ginja *et al*.^10^
Iberian	Portugal	Minhota	75	PT_MIN	50	Martínez *et al*.^16^	15	Ginja *et al*.^10^	28	Ginja *et al*.^10^
Iberian	Portugal	Mirandesa	76	PT_MIR	54	Martínez *et al*.^16^	16	Ginja *et al*.^10^	23	Ginja *et al*.^10^
Iberian	Portugal	Preta	77	PT_PRE	60	Martínez *et al*.^16^	16	Ginja *et al*.^10^	29	Ginja *et al*.^10^
Iberian	Portugal (Azores Islands)	Ramo Grande	78	PT_RGD	44	Martínez *et al*.^16^	16	Ginja *et al*.^10^	18	Ginja *et al*.^10^
Iberian	Spain	Alistana	43	ES_ALS	50	Martínez *et al*.^16^	15	This study	20	This study
Iberian	Spain	Asturiana de las Montañas	47	ES_ASM	50	Martínez *et al*.^16^	16	This study	24	This study
Iberian	Spain	Asturiana de los Valles	46	ES_ASV	50	Martínez *et al*.^16^	15	This study	25	This study
Iberian	Spain	Avileña	50	ES_AVI	50	Martínez *et al*.^16^	13	This study	18	This study
Iberian	Spain	Berrenda en Colorado	57	ES_BCO	40	Martínez *et al*.^16^				
Iberian	Spain	Berrenda en Negro	58	ES_BNE	30	Martínez *et al*.^16^	16	This study	8	This study
Iberian	Spain	Betizu	40	ES_BET	49	Martínez *et al*.^16^	15	This study	16	This study
Iberian	Spain	Bruna de los Pirineos	55	ES_BRP	50	Martínez *et al*.^16^	8	This study		
Iberian	Spain	Marismeña	59	ES_MAR	50	Martínez *et al*.^16^	16	Ginja *et al*.^10^	21	Ginja *et al*.^10^
Iberian	Spain	Monchina	41	ES_MON	50	Martínez *et al*.^16^	26	This study	18	This study
Iberian	Spain	Morucha	49	ES_MOR	50	Martínez *et al*.^16^			18	This study
Iberian	Spain	Negra Andaluza	61	ES_NAN	50	Martínez *et al*.^16^	15	This study	12	This study
Iberian	Spain	Pajuna	60	ES_PAJ	38	Martínez *et al*.^16^				
Iberian	Spain	Parda de Montaña	54	ES_PMO	50	Martínez *et al*.^16^	26	This study	25	This study
Iberian	Spain	Pasiega	56	ES_PAS	50	Martínez *et al*.^16^	14	This study	21	This study
Iberian	Spain	Pirenaica	51	ES_PIR	50	Martínez *et al*.^16^	21	This study	25	This study
Iberian	Spain	Retinta	48	ES_RET	50	Martínez *et al*.^16^	9	This study	20	This study
Iberian	Spain	Rubia Gallega	52	ES_RGA	50	Martínez *et al*.^16^	14	This study	20	This study
Iberian	Spain	Sayaguesa	44	ES_SAY	48	Martínez *et al*.^16^	14	This study	17	This study
Iberian	Spain	Serrana de Teruel	53	ES_STE	50	Martínez *et al*.^16^	18	This study	16	This study
Iberian	Spain	Lidia	42	ES_TDL	50	Martínez *et al*.^16^	72	Cortés *et al*.^63^	54	Cortés *et al*.^64^
Iberian	Spain	Tudanca	45	ES_TUD	50	Martínez *et al*.^16^	14	This study	19	This study
Iberian	Spain (Balearic Islands)	Mallorquina	63	ES_MAL	50	Martínez *et al*.^16^	15	This study	6	This study
Iberian	Spain (Balearic Islands)	Menorquina	62	ES_MEN	50	Martínez *et al*.^16^	19	This study	25	This study
Iberian	Spain (Canary Islands)	Vaca Canaria	64	ES_VCA	50	Martínez *et al*.^16^	15	Ginja *et al*.^10^	14	Ginja *et al*.^10^
Iberian	Spain (Canary Islands)	Vaca Palmera	65	ES_PAL	50	Martínez *et al*.^16^	14	Ginja *et al*.^10^	25	Ginja *et al*.^10^
British	UK (sampled in Argentina & USA)	Aberdeen Angus	79	UK_AAN	62	Martínez *et al*.^16^	25	Ginja *et al*.^10^	41	Ginja *et al*.^10^
British	UK (sampled in Argentina, Mexico, USA)	Hereford	81	UK_HER	88	Martínez *et al*.^16^	22	Ginja *et al*.^10^	45	Ginja *et al*.^10^
British	UK (sampled in USA)	British White Cattle	80	UK_BWC	30	Martínez *et al*.^16^	10	Ginja *et al*.^10^	21	Ginja *et al*.^10^
British	UK (sampled in USA)	Dexter	84	UK_DEX	43	This study	17	This study	23	This study
British	UK (sampled in USA)	Jersey	82	UK_JER	20	Martínez *et al*.^16^	18	Ginja *et al*.^10^	20	Ginja *et al*.^10^
British	UK (sampled in USA)	Shorthorn	83	UK_SHO	28	Martínez *et al*.^16^	9	Ginja *et al*.^10^	25	Ginja *et al*.^10^
Continental European	France (sampled in Portugal)	Charolais	86	EU_CHA	58	Martínez *et al*.^16^	14	Ginja *et al*.^10^	13	Ginja *et al*.^10^
Continental European	France (sampled in Portugal)	Limousin	88	EU_LIM	47	Martínez *et al*.^16^	16	Ginja *et al*.^10^	17	Ginja *et al*.^10^
Continental European	Germany (sampled in USA)	Gelbvieh	90	EU_GEB	26	This study			26	This study
Continental European	Switzerland (sampled in Mexico)	Brown Swiss	85	EU_BWS	29	Martínez *et al*.^16^	9	This study	5	This study
Continental European	Switzerland (sampled in USA)	Simmental	89	EU_SIM	19	This study			18	This study
Continental European	The Netherlands (sampled in Portugal)	Holstein-Friesian	87	EU_HOL	50	Martínez *et al*.^16^	16	Ginja *et al*.^10^	27	Ginja *et al*.^10^
African	Angola	Angola	94	AF_ANG	29	This study	19	This study	5	This study
African	Egypt	Baladi	91	AF_BAL	101	This study	16	This study	2	This study
African	Egypt	Damiata		AF_DAM					2	This study
African	Egypt	Menoufis	92	AF_MNF	27	This study				
African	Guinea	Bafatá	95	AF_BAF	20	This study	13	This study	8	This study
African	Guinea	Gabú	96	AF_GAB	25	This study	23	This study	11	This study
African	Kenya	Eastern Shorthorn Zebu	100	AF_ESZ	47	This study	25	This study	24	This study
African	Kenya	Pokot	99	AF_POK	104	This study				
African	Lake Victoria (sampled in the USA)	Ankole-Watusi	97	AF_AWA	46	This study	15	This study	13	This study
African	Mozambique	Angone		AF_AGE					15	This study
African	Mozambique	Landim	93	AF_LAN	13	This study	8	This study	18	This study
African	Mozambique	Tete		AF_TET					2	This study
African	Nigeria	Kuri	104	AF_KUR	21	This study				
African	Nigeria	Muturu	103	AF_MUT	21	This study				
African	Nigeria	Red Bororo	102	AF_RBN	14	This study	22	This study	5	This study
African	Nigeria	Sokoto Gudali	101	AF_SGN	22	This study	20	This study	9	This study
African	South Africa (sampled in Argentina)	Bonsmara		AF_BNS					11	This study
African	Zambia	Sanga Tonga	98	AF_STO	36	This study				
Indicine	India (sampled in Brazil)	Guzerat	108	IN_GUZ	15	Martínez *et al*.^16^	10	This study	20	This study
Indicine	India (sampled in Brazil)	Nelore	109	IN_NEL	89	Martínez *et al*.^16^	16	This study	27	This study
Indicine	India (sampled in Brazil)	Sindi	107	IN_SIN	11	Martínez *et al*.^16^	11	This study	1	This study
Indicine	India (sampled in Mexico & USA)	Brahman	106	IN_BRH	41	Martínez *et al*.^16^	20	Ginja *et al*.^10^	8	Ginja *et al*.^10^
Indicine	India (sampled in Mexico)	Gyr	105	IN_GYR	36	Martínez *et al*.^16^	9	Ginja *et al*.^10^	41	Ginja *et al*.^10^
**TOTAL**		**114**			**4622**		**1470**		**1797**	

Country of sample origin, breed names, numeric codes and acronyms are shown, as well as sample sizes (N). Source of reference data: (1) this study; (2) Martínez *et al*.^16^; (3) Ginja *et al*.^10^; (4) Cortés *et al*.^63^; (5) Cortés *et al*.^64^; (6) Egito *et al*.^28^; (7) Vargas *et al*. ^65^.

### Genetic diversity

Overall, the genetic diversity of Creole cattle was high across all markers (Table 2). Mitochondrial DNA variation was inferred from roughly 700 bp D-loop sequences in 1,470 animals across 93 breeds. Maternal genetic diversity measured as haplotype diversity (H) and total number of haplotypes was greatest in Iberian (H = 0.972; No. Haplotypes = 248), Creole (H = 0.966; No. Haplotypes = 117) and African cattle (H = 0.961; No. Haplotypes = 78), whereas Continental European (H = 0.931; No. Haplotypes = 31) and British (H = 0.920; No. Haplotypes = 52) transboundary breeds had intermediate values, and Indicine cattle (H = 0.903; No. Haplotypes = 62) had the lowest haplotype diversity despite the differences in the number of sampled animals within each group. Estimates of mtDNA genetic diversity and haplogroup frequencies within cattle populations are shown in Supplementary Table S1. Among Creole breeds, the greatest diversity was observed in Cr. Poblano, Blanco Orejinegro, Cr. Chiapas and Cr. Argentino (H ≥ 0.883; No. Haplotypes ≥ 10) whereas the breeds Cr. Patagónico, Guabalá, Senepol had the lowest estimates (H ≤ 0.694; No. Haplotypes ≤ 5). The African breed with the greatest maternal diversity was Baladi (H = 0.933; No. Haplotypes = 15) and Ankole Watusi the lowest (H = 0.667; No. Haplotypes = 5).

**Table 2:** Number of breeds/animals analyzed and genetic diversity indicators for the various breed groups, inferred from mitochondrial DNA (mtDNA), Y-chromosome (Ychr) and autosomal microsatellite (MS) data.

Genetic marker	Item		Creole	Iberian	British	Continental	African	Indicine	Global
mtDNA	No. Breeds		33	36	6	4	9	5	93
	No. Animals		460	627	101	55	161	66	1470
	Haplotype diversity		0.966	0.972	0.920	0.931	0.961	0.903	0.942
	No. Haplotypes		117	248	52	31	78	27	463
	Haplogroup frequency	T	0.000	0.000	0.010	0.000	0.012	0.000	0.002
		T2	0.009	0.021	0.000	0.018	0.025	0.000	0.015
		T3	0.713	0.868	0.990	0.982	0.050	0.500	0.726
		Q	0.030	0.010	0.000	0.000	0.000	0.000	0.014
		T1	0.165	0.093	0.000	0.000	0.832	0.121	0.188
		T1c1a1	0.083	0.010	0.000	0.000	0.081	0.364	0.055
		I	0.000	0.000	0.000	0.000	0.000	0.015	0.001
Ychr	No. Breeds		31	36	6	6	13	5	97
	No. Animals		520	774	175	106	125	97	1797
	Haplotype diversity		0.884	0.790	0.575	0.421	0.842	0.435	0.658
	No. Haplotypes		21	20	7	5	25	2	58
	Haplogroup frequency	Y1	0.350	0.292	0.857	0.264	0.088	0.000	0.332
		Y2	0.254	0.708	0.143	0.736	0.424	0.000	0.465
		Y3	0.396	0.000	0.000	0.000	0.488	1.000	0.203
MS	No. Breeds		39	39	6	6	14	5	109
	No. Animals		1474	1930	271	229	526	192	4622
	Genetic diversity	H_e_	0.809 (0.014)	0.772 (0.020)	0.755 (0.015)	0.758 (0.020)	0.790 (0.017)	0.698 (0.024)	0.763 (0.008)
		N_a_	15.5 (0.9)	12.9 (0.8)	9.5 (0.5)	10.6 (0.8)	14.0 (0.8)	11.2 (0.7)	12.3 (0.4)
		N_e_	5.8 (0.5)	4.9 (0.4)	4.4 (0.3)	4.6 (0.4)	5.2 (0.3)	3.8 (0.4)	4.8 (0.2)

Details on the breeds included in each geographic group are in Table 1. For mitochondrial DNA, the total number of haplotypes and haplotype diversities were estimated for a 700 bp D-loop region, and animals/breeds with incomplete sequence data were only used for haplogroup assignment. Genetic diversity indicators for autosomal microsatellites correspond to expected heterozygosity (He), mean number of alleles/locus (Na) and effective number of alleles/locus (Ne) with standard deviation in ().

The 58 Y chromosome haplotypes found in 1,797 bulls across 97 breeds were defined using a combination of one indel, one SNP and 5 microsatellite loci (ZFY10-UTY19-DDX3Y1-BM861-INRA189-UMN0103-UMN0307). Y chromosome haplotype diversity was highest in Creole cattle (H = 0.884; No. Haplotypes = 21), followed by African (H = 0.842; No. Haplotypes = 25) and Iberian (H = 0.790; No. Haplotypes = 20) breeds. Indicine cattle had the lowest paternal diversity (H = 0.435; No. Haplotypes = 2). Y-chromosome haplotype and haplogroup frequencies observed in each breed, as well as estimated haplotype diversities per breed are detailed in Supplementary Table S2. The Creole breeds showing the highest Y-chromosomal diversity were Cr. Chiapas, Cr. Lageano, and Cr. Chihuahua breeds (H ≥ 0.750; No. Haplotypes ≥ 4) with the three major Y-haplogroups represented. The lowest paternal diversity was observed in Pampa Chaqueño, Senepol, Guaymí, and Cr. Limonero breeds (H ≤ 0.227; No. Haplotypes ≤ 2) which belong mainly to a single Y-haplogroup. Within African cattle, Landim and Gabú breeds had the greatest Y-chromosomal diversity (H ≥ 0.810; No. Haplotypes ≥ 8) with three and two haplogroups represented, respectively, and the breeds Ankole Watusi, Damiata, Red Bororo and Sokoto Gudali were fixed for a single haplotype.

For microsatellite loci, the Creole breed group showed the highest levels of genetic diversity for all the parameters estimated, namely Nei’s unbiased gene diversity (He), mean number of alleles (Na) and effective number of alleles (Ne) (He = 0.809 ± 0.014; Na = 15.5 ± 0.9; Ne = 5.8 ± 0.5), followed by the groups of African (He = 0.790 ± 0.017; Na = 14 ± 0.8; Ne = 5.2 ± 0.3) and native Iberian (He = 0.772 ± 0.020; Na = 12.9 ± 0.8; Ne = 4.9 ± 0.4) native breeds. Estimates of genetic diversity determined in each breed based on microsatellite data are shown in Supplementary Table S3. The Creole breeds with the highest autosomal diversity were Suriname, Cr. Nayarit, Caqueteño, Hartón del Valle and Cr. Chiapas (He ≥ 0.782; Na ≥ 7.53; Ne = 4.60), whereas Guabalá, Romosinuano and Cr. Patagónico had the lowest levels of genetic diversity (He ≤ 0.670; Na ≤ 5.79; Ne ≤ 3.53).

### Phylogenetic relationships

**Figure 1:**
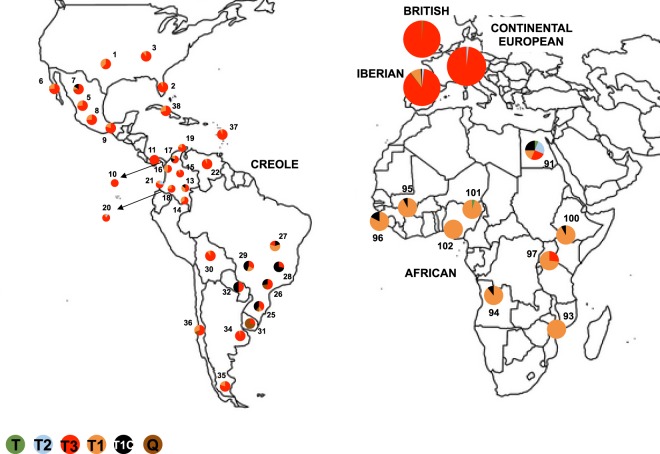
Geographic and breed distribution of maternal haplogroups in Creole and African cattle. For the Iberian, British, and Continental European cattle, haplogroup frequencies are summarized for each group. The Indicine cattle included in our study were sampled in the American Continent and are not shown in the figure. Different colors indicate major mitochondrial haplogroups and numbers in the figure correspond to the breed codes defined in Table 1. Detailed information on the mitochondrial diversity found in each breed is in Supplementary Table S1.

Geographic and breed distribution of maternal haplogroups are depicted in Fig. 1 and Supplementary Table S1. Overall, the most frequent maternal lineages were the European T3, namely in Iberian (over 86%), British (99%) and Continental European (~98%) cattle, but also in Creole (~71%) and Indicine (~50%) breeds. T and T2 lineages were somewhat residual, while the more distinct Q-haplogroup was found exclusively in Creole and Iberian cattle (less than 3%). Most African cattle belong to the T1 haplogroup (~83%) which is also found within the Creole (~16%) and Iberian (~9%) breed groups. Interestingly, the T1c-haplogroup is equally observed in Creole and African breeds (~8%) while it is mostly residual in Iberian cattle or absent in other European breeds. Note that the Indicine breeds of the Americas sampled in our study essentially carry taurine mitochondrial DNA (including the African T1 and T1c lineages, at frequencies of ~12% and ~36%, respectively) and only one animal of the Guzerat breed had the I haplogroup which is typical of Indian cattle.

**Figure 2:**
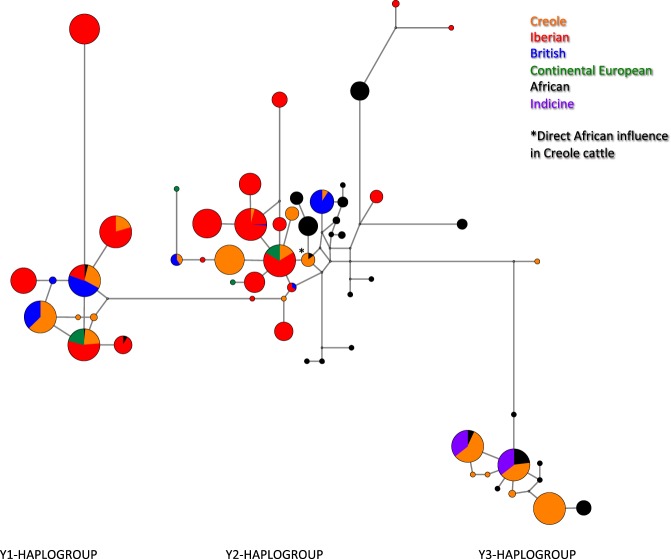
Median-Joining network representing genetic relationships between the Y-chromosome haplotypes observed across geographic breed groups and within each major haplogroup (Y1, Y2 and Y3). Colors represent the geographic origin. Further details on the haplotype diversity observed in each breed are shown in Supplementary Table S2.

We observed 11 and 35 haplotypes of the Y1 and Y2 haplogroups, respectively, which are characteristic of taurine cattle, whereas 12 haplotypes were of the typical indicine Y3-haplogroup (Supplementary Table S2). The Y1-249-158-98-124-151 haplotype was the most frequent within the Y1 haplogroup, particularly in British cattle. While African cattle contained 14 unique Y2-lineages, the Y2-249-158-102-132-149 haplotype was the most common within the Y2-haplogroup mainly in Iberian breeds. Among indicine cattle, the Y3-245-156-88-116/124-149 haplotype was the most common, but five novel Y3 haplotypes were also detected in African breeds (namely in the Eastern Shorthorn Zebu cattle from Kenya). The Caracú and Mocho Nacional breeds from Brazil are mostly fixed for a highly distinct patriline (Y3-245-156-90-114/124-151 haplotype) not found elsewhere. This is also true for Guabalá and Jersey, each fixed for a unique lineage within the Y2-haplogroup (Y2-245-158-102-132-149 and Y2-249-158-104-128-149, respectively). Phylogenetic relationships among Y-chromosome haplotypes and haplogroups are shown in the Median-Joining Network of Fig. 2. Creole cattle were the most heterogeneous with all three haplogroups represented at somewhat similar frequencies. Y3 and Y2 lineages were almost equally represented in African cattle, and Y1 haplotypes were below 9%. Iberian and Continental European breeds mainly belong to the Y2 haplogroup (over 70%), whereas commercial transboundary British breeds carry Y1 lineages (over 85%).

**Figure 3:**
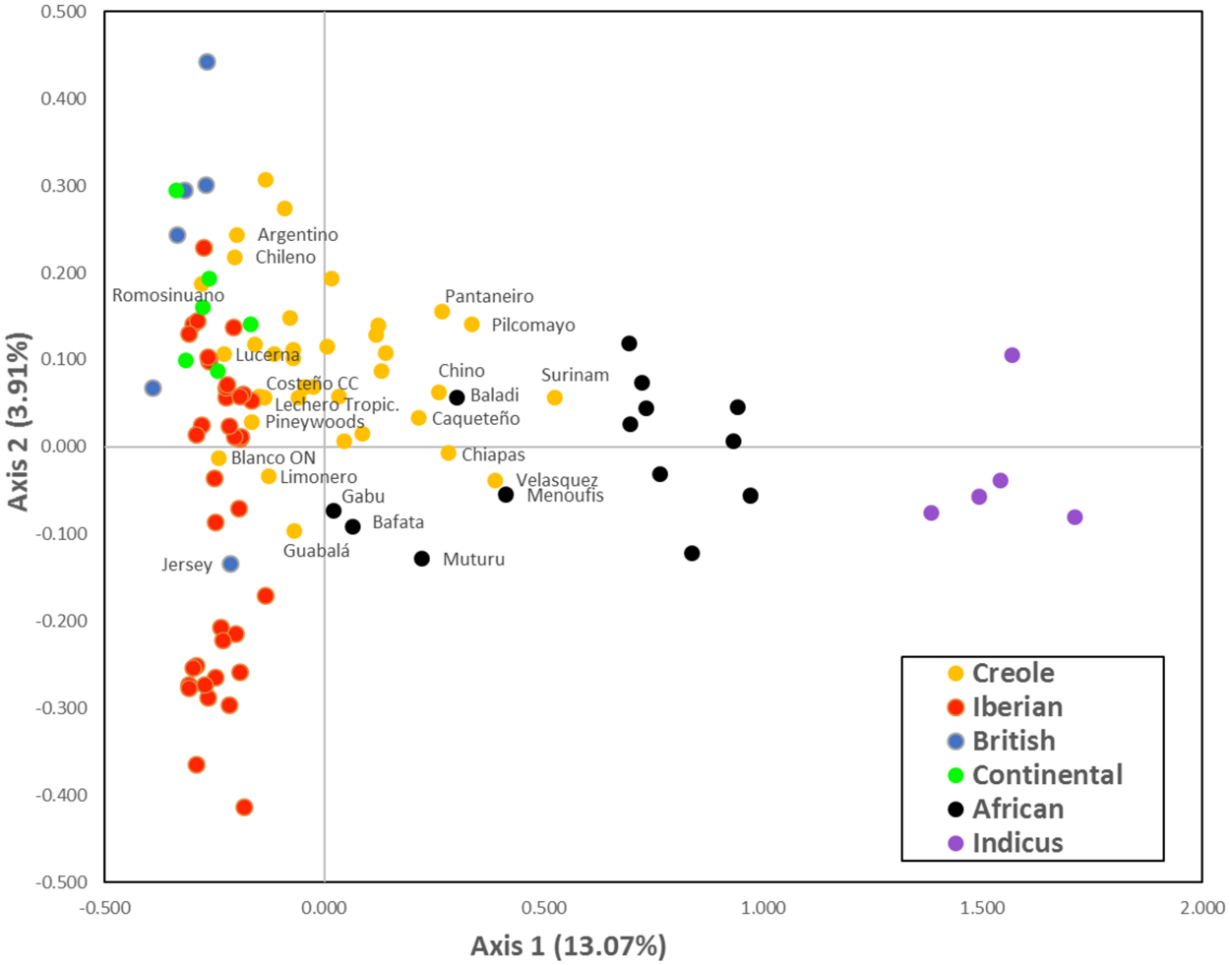
Spatial representation of genetic distances among the breeds analyzed, from the first two axes obtained in the factorial analyses of correspondence based on microsatellite data. Values between brackets on both axes represent the contribution in % of each axis to total inertia. Colors represent the geographic origin as shown in the figure. The names of some breeds that correspond to areas of overlapping between groups are also shown.

In the Factorial Correspondence Analysis obtained with autosomal microsatellite genotyping data, the first and second axes accounted for about 13% and 4% of the total variability, respectively. The two-dimensional plot of breed coordinates defined by the first two major axes is in Fig. 3, excluding the two Cuban Creoles and the Spanish Sayaguesa, as these were outliers in the distribution. The 106 breeds represented spread along the first axis according to their continent of origin, with a remarkable separation between indicine and taurine breeds. The Creole breeds showed no discontinuity relative to Iberian breeds on one side and to African breeds on the other. The African Gabú and Bafata breeds from Guinea-Bissau, the Muturu from Nigeria and the Baladi and Menoufis from Egypt revealed a closer relationship with the Creole breeds, in particular with the Surinam Creole, the Velasquez, Caqueteño and Chino Santandereano from Colombia, the Pantaneiro from Brazil, the Pilcomayo from Paraguay and the Guabalá from Panama. On the other hand, some Creole breeds showed a closer relationship with Iberian breeds, particularly the Creoles from Argentina and Chile, the Romosinuano, Lucerna, Costeño con Cuernos and Blanco Orejinegro from Colombia, the Criollo Lechero Tropical from Mexico, the Limonero from Venezuela and the Pineywoods from the United States. The second axis in the Factorial Correspondence Analysis accounted for nearly 4% of the variance and essentially resulted in the spreading of European breeds along this axis, with a visible separation of Iberian relative to the Continental and British cattle breeds. One interesting exception was the Jersey, which clearly separated from the other British breeds.

**Figure 4:**
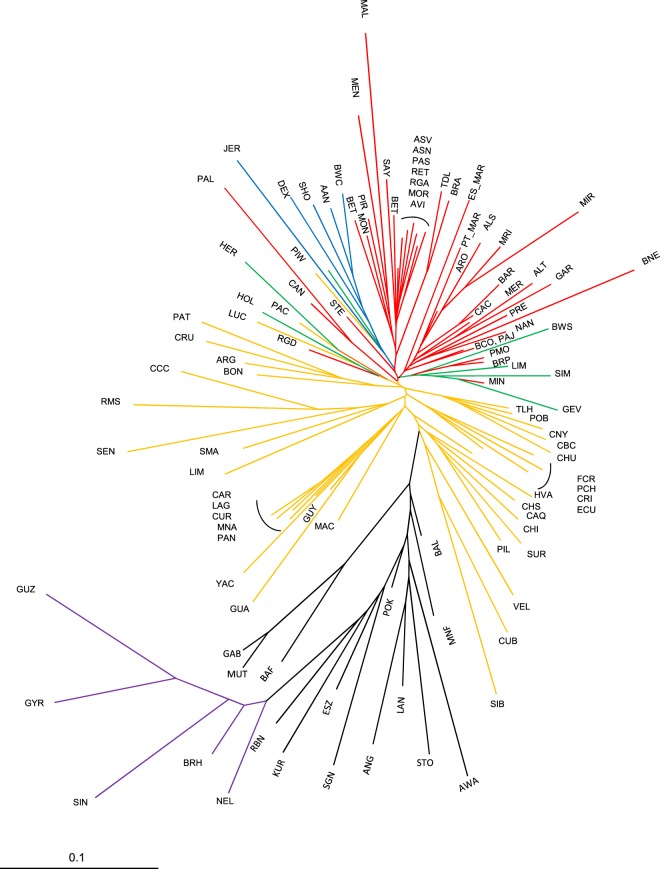
Neighbour-joining tree representation of Nei’s DA genetic distances between 109 breeds based on microsatellite data, with colors representing geographic breed groups as defined in Fig. 3. Breed acronyms are as defined in Table 1.

The neighbor-joining tree representing DA genetic distances between breeds (Fig. 4) reveals a continental clustering of the breeds evaluated, with Creole breeds placed between the African and European (including Iberian) clusters. The confidence levels of the relationships between breeds was inferred by the bootstrap values (Supplementary Fig. S1) which were generally low for the nodes close to the root but tended to be higher when smaller groups of breeds were evaluated.

Using autosomal microsatellites, the vast majority of the Iberian breeds clustered together, but a few showed signs of exotic admixture (Ramo Grande, Minhota, Bruna de los Pirineos, Parda de Montaña and Serrana de Teruel) and grouped with the corresponding British or Continental European breeds. Nearly all the British breeds clustered together, and the same happened with Continental European breeds. The Creole breeds essentially clustered according to their geographic origin, with clades broadly corresponding to: (1) North American and Mexican breeds; (2) breeds from Argentina and Uruguay; (3) the majority of Colombian breeds; (4) breeds from Brazil and Panama; (5) breeds from Cuba; (6) one large cluster representing diverse geographic origins, including Chiapas, Ecuador, Paraguay and some Colombian breeds. African breeds had a common root diverging from Creoles, and several clusters could be identified, mostly reflecting the geographic origin of the breeds analyzed. These clusters included: (1) breeds from the West Coast of Africa (Bafata and Gabú from Guinea-Bissau and Muturu from Nigeria); (2) remaining breeds from Nigeria (Kuri, Sokoto Gudali and Red Bororo) and the Pokot and Eastern Shorthorn Zebu from Kenya; (3) Baladi and Menoufis breeds from Egypt; (4) cluster of breeds from the southern part of Africa, including the Ankole-Watussi, Sanga Tonga from Zambia, Landim from Mozambique and taurine cattle from Angola. The last major branch in the dendrogram grouped all the zebu breeds represented in our study, which showed an important genetic differentiation from each other and diverged from the group of African breeds.

The low bootstrap values observed for large breed-groups could be anticipated, as this is the pattern expected when many populations are analyzed^21^, particularly if they represent closely related breeds^9,22–24^. Nevertheless, the general clustering of breeds from Nei’s genetic distances was consistent with the results obtained with other methodologies such as Factorial Analysis of Correspondence and the Bayesian approach implemented with Structure. When the tree of genetic distances between breed groups is considered (Supplementary Fig. S2) the bootstrap values indicate a very strong separation, with the Creoles occupying an intermediate position between the Iberian and African groups, while the latter are positioned between the European and indicine groups.

### Model-based clustering/Genetic structure

**Figure 5:**
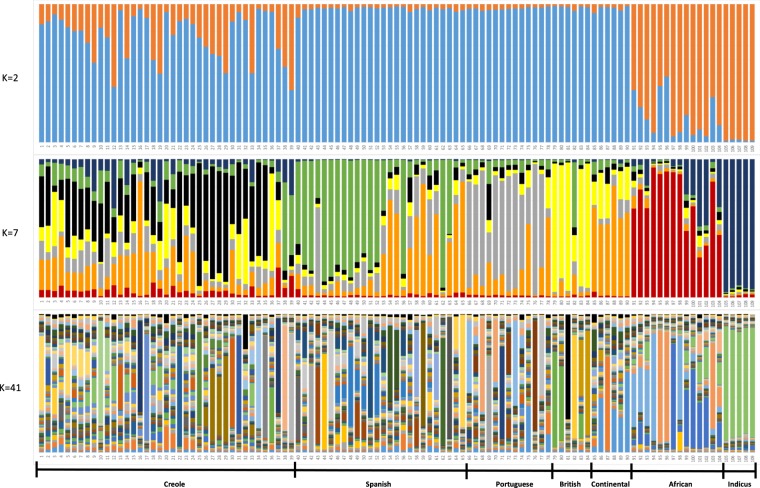
Population structure of 109 cattle breeds inferred by using the STRUCTURE software and based on microsatellite data. Each breed is represented by a single vertical bar divided into K colors, where K is the number of assumed ancestral clusters, which is graphically represented for K = 2, 7 and 41. The colored segment shows the breed’s estimated membership proportions in a given cluster. Breed numerical codes are as defined in Table 1. Ancestral contributions for other values of K ranging from K = 2 to 40 are shown in Supplementary Fig. 2.

A Bayesian clustering approach was used to assess breed structure and relationships using autosomal microsatellite data, assuming that the observed genetic diversity results from the genetic contributions of a variable number of ancestral populations (K). Contributions of the assumed ancestral populations to each one of the 109 breeds studied are presented in Fig. 5, for representative values of K = 2,7 and 41 (for values of K = 2 to K = 40 such contributions are shown in Supplementary Fig. S3). When K = 2 was assumed, the European breeds (Spanish, Portuguese, British and Continental) separated from the Indicus group, and most African breeds revealed proximity with the latter group. On the other hand, the American Creoles showed evidence of mixed ancestry from various sources, even though for most Creole breeds the major contribution was from the European group.

When the number of ancestral populations was assessed at K = 7 (i.e., the number of breed groups considered in our data set), the Indicine and African groups separated clearly, even though there were signs of indicine admixture in some African breeds, especially those from Kenya and Nigeria. The British breeds represented a very homogeneous group, with the exception of the Jersey, which was rather different and showed some similarity with a few of the Spanish breeds. The continental breeds mostly clustered together, but a few of them revealed some proximity with British breeds and some had similarity with a few Spanish and Creole breeds. The Portuguese breeds were fairly homogeneous, with the exception of two breeds (Ramo Grande and Minhota) which had clear signs of admixture with Continental European breeds. The Spanish breeds clustered in two groups, the first corresponding essentially to the group of Red breeds which share a common origin, while the second group includes the majority of the southern Spanish breeds and show some similarity with Continental European breeds. The Creole group is the one showing a more diversified background, with contributions from all the other groups represented in nearly all Creoles. Nevertheless, most Creole breeds share a distinct common ancestry, which spreads across the Americas and is mostly perceptible in Creole breeds from the United States, Mexico, Panama, Colombia, Venezuela, Brazil, Uruguay, Paraguay and Argentina. All Creole breeds displayed a minor relationship with African cattle, particularly noticeable in Caribbean cattle (Senepol and Siboney). On the other hand, an indicine contribution was detectable in many Creoles, especially the Caqueteño and Velasquez breeds from Colombia, the Creole breeds from Cuba and Suriname, and the Mexican Criollo from Chiapas. Admixture with British breeds was detectable in the Pineywood from the United States, Lucerna from Colombia, Pampa Chaqueño from Paraguay and the Creoles from Uruguay and Chile. The sharing of ancestry with the Portuguese group was detectable in all Creoles, but more noticeable in the Chiuahua and Nayarit from Mexico, the two Creoles from Panama and the Blanco Orejinegro, Sanmartinero and Costeño con Cuernos from Colombia. The Creoles from the United States, Ecuador, Bolivia, the Criollo Lechero Tropical from Mexico and the Majority of the Colombian Creoles shared an influence, which could be either from cattle breeds from southern Spain or from the other Continental European breeds.

The most likely number of ancestral populations, assessed by the method of Evanno *et al*.^25^ was K = 41. When this large number of ancestral populations was evaluated, very heterogeneous results were obtained for the majority of the breeds studied, even though some interesting details could be identified. The indicus breeds were quite homogeneous, with little indication of introgression from other breeds, while the majority of the breeds of the African group did not reveal major signs of admixture with indicus. The African group was clearly subdivided, with one cluster made up by the two breeds from Guinea-Bissau (Bafata and Gabú) and the Muturu from Nigeria, another cluster formed by Ankole-Watusi and the breeds from Kenia and Nigeria, and one last cluster represented by the breeds of Egypt, Angola, Mozambique and Zambia. The Continental European breeds resulted, in general, from the contribution of several ancestral populations, with the exception of the Holstein which essentially represented one ancestral population. In the British group, the Hereford and the Jersey were isolated from the other breeds, while the remaining breeds were grouped in two clusters, one formed by Angus and White Cattle, and the other by Dexter and Shorthorn. The Portuguese breeds had heterogeneous contributions from various ancestral populations, which generally differed from one breed to another. Nevertheless, some breeds that are known to have a common phylogenetic relationship or a close geographic distribution displayed some similarity. For the group of Spanish breeds, a few of them showed a diversified and heterogeneous ancestry, but most breeds clustered independently, possibly reflecting the influence from a specific ancestral population, and this pattern was found both in highly threatened breeds (such as the Palmera and Menorca breeds) as well as in breeds with large census (such as the Retinta and the Lidia). For Creole breeds the pattern was generally very distinct from one breed to another, strongly supporting the uniqueness of the various Creole breeds. For example, The Costeño con Cuernos, Limonero, Caracú, Yacumeño, Uruguayo, Argentino, Patagónico, Chileno and Senepol all had strong individuality, with a major contribution of each one’s own ancestral population. On the other hand, a few breeds with a close geographic distribution showed common ancestry, and this was the case for the Brazilian cluster (Crioulo Lageano, Curraleiro, Mocho Nacional and Pantaneiro), the Panamanian group (Guabala and Guaymí) and the North American group (Texas Longhorn and the majority of Mexican breeds). At this high level of K, no clear evidence could be found in American Creoles of admixture with any of the other breed groups evaluated, possibly with the exception of an indicine contribution to a few Creole breeds.

## Discussion

We investigated the genetic diversity, uniqueness and population structure of Creole cattle using molecular markers. The results of our combined analysis of uniparental mitochondrial DNA and Y-chromosome markers with autosomal microsatellite data are highly consistent in showing the heterogeneous origins of Creole cattle from the Americas, but also to support the fact that Creole breeds are distinct entities, which demands for in-depth research to have a better knowledge of their characteristics. Historic admixture is reflected in their extremely high genetic diversity for maternal (H = 0.972; No. Haplotypes = 248), paternal (H = 0.884; No. Haplotypes = 21) and autosomal (He = 0.809; Na = 15.5; Ne = 5.8) estimates. These results are consistent with previous studies in smaller subsets of Creole breeds using classic genetic markers^8,10,15,26–29^.

The distribution of genetic diversity varies widely among Creole breeds from the different countries. In general, Creole breeds from Mexico (e.g. Cr. Chiapas, Cr. Chihuahua, Cr. Nayarit) and USA (e.g. Florida Cracker) showed high genetic diversity across markers, whereas breeds from the Caribbean region (e.g. Senepol, Guaymí, Guabalá) had lower values. This scenario may reflect the threatened status of some Creole cattle populations, the former due to dilution from intensive crossbreeding and the latter as a consequence of isolation and abandonment. Creole cattle represent an enormous reservoir of genetic diversity for the species, despite the fact that many of these breeds are on the brink of disappearance^30^. There is now an increased interest in maintaining these important animal genetic resources. Within Red Conbiand and the BioBovis consortium, researchers have contributed significantly to increase awareness on this matter and several Creole breeds now have a herdbook managed by producers’ associations or are under conservation programs with significant expansion in various countries, as has been recently reported in a survey carried out in the framework of a FAO-CONBIAND agreement^31^.

African cattle also retain high genetic diversity probably due to less intensive management. In particular, we identified 14 and five unique Y2 and Y3 lineages, respectively. The majority of the novel Y2-diversity was found in the Landim cattle from Mozambique, as well as in the Gabú and Bafatá breeds from Guinea, while the Eastern Shorthorn Zebu from Kenya accounted for four of the five new Y3-haplotypes (Supplementary Table S2). Interestingly, the sharing of Y2-249-158-102-130-149 haplotype between 6 Creole animals from Argentina (4 Cr. Argentino and 2 Cr. Patagónico) and one animal from the Landim breed from Mozambique, a former Portuguese colony, suggests a direct male influence from Africa in some Creole breeds, particularly in the southern region of South America. Additionally, other studies have shown that mitochondrial DNA sequence variation also provides support for an African maternal influence in Creole cattle of the Americas^8,10,11,32,33^, and our results appear to suggest that T1c-lineages, which are very scarce in Iberia, may have been introduced directly from Africa. Specifically, we observed these lineages in cattle from Guinea-Bissau and Angola and the possibility that cows from these two countries could be the direct sources of the T1c haplogroup detected in American Creoles in our study would indicate that cattle may have been taken aboard transatlantic slave ships, since these regions were of major historical importance as departure points of slave trade routes^34^. Furthermore, T1-lineages, which have been shown to exist in Iberia at least since Roman times^35,36^, could have been introgressed into Creole breeds either by the Iberian founder cattle during the early stages of colonization of the Americas or directly from African animals, or both.

The indicine animals included in our study represent the most common zebu breeds that expanded through the Americas over the 20th century. According to historic information, bulls from these breeds were introduced from India and backcrossed with local Creole cows^37^. The matrilines represented in American indicine cattle are expected to correspond essentially to the female population that was the foundation of this systematic backcrossing system rather than to the matrilines present in India. Thus, it is not surprising that the maternal diversity found in our indicine samples had a very scarce representation of the I-haplogroups^38^ typical of zebu cattle from India. Indeed, with the exception of one animal of the I haplogroup, we could only detect the taurine matrilines of African and Iberian origins, confirming the findings of Curi *et al*.^39^ and Ribeiro *et al*.^40^ who have reported that the vast majority of indicine cattle in Brazil carry taurine mitochondrial lineages.

Our results from autosomal microsatellites revealed a transition across continents, with more distant groups corresponding to the indicus and European clusters, and with the African group in an intermediate position. On the other hand, Creole breeds showed their own identity in most cases, but also sometimes showed detectable influences from the three groups above which differed between Creole breeds. This is in agreement with results reported for some Creole breeds in studies where SNP chip arrays were used^17,19,41^.

The indicine group of breeds essentially shared a common ancestry, even though the breeds analyzed differed considerably from each other for the panel of microsatellites studied. The African group of breeds was very diverse, with a breed structure and relationships largely reflecting their geographic distribution. Most African breeds showed some extent of indicine admixture, which was however less pronounced in the breeds from Guinea-Bissau (Bafatá and Gabú) and the Muturu from Nigeria. These breeds from the West Coast of Africa showed a close relationship. They belong to the N’Dama taurine group, which is recognized for its high resistance to trypanosomiasis, thus allowing their maintenance in Tsetse infested areas where other breeds are unable to survive^42^. Another cluster included the Ankole Watusi, Pokot and East Shorthorn Zebu from Kenya, and the Sokoto Gudali and Red Bororo from Nigeria, which present a pronounced indicine admixture, as has been shown previously^42–44^. The last group of breeds presents a different identity and occupies the Zambezian region (cattle from Angola, Landim from Mozambique and Sanga Tonga from Zambia) but it also clusters with the two breeds from Egypt (Baladi and Menoufis), possibly reflecting a common ancestry for the two groups. Some of the African breeds studied here have not been genetically characterized in the past (breeds from Angola, Mozambique, Guinea-Bissau, and Egypt), and further studies are needed to better understand their origins and relationships.

The Creole group was the main focus of our work, and it presented some peculiar features, such that most Creole breeds had their own identity or grouped with a few other Creoles with a nearby geographic distribution. In agreement with previous reports^16^ we detected a considerable diversity among the various Creole breeds analyzed, where some Creoles showed important influence from indicus (especially breeds raised in tropical areas such as the Creoles from Cuba and Suriname and some of the Creole breeds from Colombia and Mexico) while other Creoles did not. The results from AFC (Fig. 3) show that the Creole breeds occupy the center of the distribution plot, between the African and Iberian breeds. These results point in particular to a possible influence of African cattle on Creole breeds from Panama, Mexico, Colombia and Brazil, with a more likely contribution of cattle originating from Western Africa (Guinea-Bissau, Nigeria) and Northern Africa (Egypt). Other Creole breeds, especially those from Panama and Colombia, revealed signs of Iberian influence. The analyses with the model-based clustering procedures implemented by Structure (Fig. 5) assuming K = 7 confirm that most Creoles essentially have a common identity separate from the other breed groups, even though some Creole breeds reveal limited contributions from the other groups. When many ancestral populations are assumed (K = 41) some Creoles show mixed contributions from various ancestral populations, but most Creole breeds remain uniquely linked to their own single cluster or share a common ancestry with breeds in the same geographical vicinity. This was the case, for example, for the groups of breeds from Brazil, Panama and North America, which formed distinct clusters.

These results strongly support the idea that Creole breeds have their own identity and deserve to be adequately managed and conserved. Our comprehensive sampling of Creole cattle allowed us to clearly infer the influence of African and European founders, confirming observations from previous studies^7,19^, but also to better understand how breeding strategies shaped their genetic composition. In some Creole breeds the analysis with microsatellites indicates that there are still signs of an African and Iberian influence, but these signatures are not as strong as when uniparental markers are investigated. In particular, here we could identify complex patterns of male mediated gene flow through the presence of Y1, Y2 and Y3 lineages in creole breeds. Our results also confirm that more intensively managed cattle populations are typically fixed for a single patriline^45,46^, thus haplotype diversity was null in many British, Continental European and Indicine transboundary commercial breeds, but also in many local breeds from Iberia. Even though Creoles have likely originated mostly from Iberian cattle, with some additional influences from African and British cattle, the small size of the founder populations^2^ and a long process of genetic drift and adaptation to the conditions of the New World have led to the divergence of Creoles from their ancestors, resulting in populations which are currently quite distinct in most cases. These results support further analyses at the genome level to infer adaptation/selection to specific environmental and breeding conditions, and additional studies using genomic approaches are warranted, even though biased SNP chips designed for commercial breeds may be inadequate for Creoles.

## Conclusions

Our findings combining three types of genetic markers in a broad representation of cattle breeds sampled in various continents, integrated with historical information, indicate that Creole breeds have their own identity and a fingerprint unique to this group. These breeds need to be studied in greater depth to better assess their integration in sustainable rural development. The genetic legacy of Iberian cattle is still represented in Creoles, but other influences could also be detected, even though in most cases Creoles remain well differentiated. The African contribution to the genetic composition of Creoles is clear in our work, and while in some cases this may occur by an indirect path through Iberian breeds, the direct influence of African breeds on Creole cattle is undoubtedly demonstrated by their sharing of unique maternal and paternal lineages. Programs aimed at the genetic management of Creole breeds of cattle are urgently needed, aimed at the characterization, conservation and valuation of these unique genetic resources. With this goal, efforts must be made to overcome the gap existing between the state-of-the-art genomic tools currently available and their application to local breeds, especially in the case of undervalued breeds kept in marginal regions such as Creoles^47^.

## Methods

### Ethics statement

Biological samples were collected during routine veterinary checkups in the framework of official health control programs and with the agreement of breeders.

### Sample collection and microsatellite genotyping

We studied a total of 4,658 animals from 114 cattle breeds, including 1,480 Creole from 40 breeds, 1,930 Iberian from 39 breeds, 556 African from 18 breeds, 271 British from 6 breeds, 229 Continental European from 6 breeds, and 192 Indicine from 5 breeds (Table 1). The sampling strategy was designed in the context of the BioBovis Consortium (https://biobovis.jimdo.com/) to cover the wide geographic range of dispersal of Creole cattle. Breeds from other regions were included as well to capture historical signatures of cattle introductions into the Americas. Blood, semen or hair root samples were collected by qualified veterinarians during their routine practice in the framework of official health control programs. Therefore, no ethical approval was required for sampling of biological material. To minimize the degree of relationship among individuals, unrelated animals from different herds were selected whenever possible. Genomic DNA was isolated as previously described^10,48^ following routine procedures. A set of 19 microsatellite markers was selected according to the recommendations of the Food and Agriculture Organization of the United Nations (FAO) and the International Society for Animal Genetics^49^ for genetic diversity studies in cattle. Amplification in multiplex PCRs and genotyping conditions were as in Martínez *et al*.^16^. Allele sizing was standardized as detailed in Delgado *et al*.^50^. Genotypes for the 109 breeds analyzed are accessible via the Dryad repository.

### Mitochondrial DNA sequencing

The maternal lineages of cattle were examined in a subset of 1,470 animals from 93 breeds, including 460 Creole from 33 breeds, 627 Iberian from 36 breeds, 161 African from 9 breeds, 101 British from 6 breeds, 55 Continental European from 4 breeds, and 66 Indicine from 5 breeds (Table 1). In cattle mitochondrial DNA (mtDNA) haplogroups are geographically structured and the hypervariable D-loop fragment analyzed (*Bos taurus* reference sequence NCBI accession number V00654, between bases 8–169 and 16,050–16,302) allows to identify major maternal lineages as defined by mitogenomes. Amplification conditions and sequencing were done as described in Ginja *et al*.^10^. Products were separated on ABI 3730 DNA Analyzer instruments (Applied Biosystems) and sequences analyzed with SEQMANTM II v6.1 (DNASTAR Inc.). Sequences of the D-Loop fragment analyzed in this study (Table 1) are accessible in the European Nucleotide Archive (ENA) web page, accession numbers ERS3397191- ERS3398032.

### Y chromosome

Y chromosome haplotype analysis was done using a subset of 1,797 animals from 97 breeds, including 520 Creole from 31 breeds, 774 Iberian from 36 breeds, 125 African from 13 breeds, 175 British from 6 breeds, 106 Continental European from 6 breeds, and 97 Indicine from 5 breeds (Table 1). We used a combination of Y-specific markers to investigate paternal variation and depict major Y-haplogroups found in cattle. Genotyping conditions for one indel (ZFY10), one SNP (UTY19) and five STRs (DDX3Y1, BM861, INRA189, UMN0103 and UMN0307) were as previously described^46,51^. For some animals, the USP9Y marker was also genotyped for haplogroup confirmation following Bonfiglio *et al*.^52^.

### Statistical analyses

#### Genetic diversity

We estimated genetic diversity parameters for each breed and geographic breed group using mitochondrial DNA, Y chromosome and microsatellite data with GENALEX v6.0^53^. For mitochondrial and Y-chromosome markers, haplotypes were identified and the frequency of each haplogroup was determined, as well as haplotype diversity (H). The total number of mtDNA haplotypes and mtDNA haplotype diversities were estimated for a 700 bp D-loop region, animals/breeds with incomplete sequence data were only used for haplogroup assignment. For microsatellite markers, we estimated the unbiased Nei’s heterozygosity (He), the total number of alleles (Na) and the effective number of alleles (Ne). Detailed information on the breeds included in each analysis can be found in Supplementary Materials (Supplementary Tables S1–S3).

#### Genetic relationships

Phylogenetic relationships among Y-chromosome haplotypes were investigated using the median-joining (MJ) network method^54^ implemented in NETWORK v5.0.0.3 (Fluxus Technology Ltd, Suffolk, England, 2004–2018). Details of the phylogenetic analyses can be found in our previous publications^10,46^. Haplotype components were weighted so that the locus with the lowest expected mutation rate was assigned the highest weight^55^. For microsatellite loci, pairwise Nei’s DA distances^56^ between breeds or geographic breed groups were calculated in Populations software^57^ and used to obtain a Neighbor-Joining dendrogram to depict genetic relationships. Bootstrap values were obtained with 1,000 replicates over loci. Also, FSTAT v. 2.9.3^58^ was used to estimate the *F*-statistics per locus according to Weir & Cockerham^59^, and P values were obtained based on 1,000 randomizations. A Factorial Correspondence Analysis to represent breed relationships based on microsatellite allele frequencies was carried out using the function ‘AFC 3D sur populations’ in GENETIX v4.04.05^60^.

#### Model-based clustering

We used multilocus microsatellite data with the STRUCTURE software^61^ to carry out a model-based clustering analysis and assign individuals to populations as described by Martínez *et al*.^16^. For each ancestral K value, we performed five independent simulations, from K = 2 to K = 112, using a burn-in of 100,000 iterations and a run length of 300,000 iterations. The parameter alpha (degree of admixture) was inferred from the data using the default settings and an admixture model with correlated allele frequencies^62^. The method of Evanno *et al*.^25^ was used to determine the modal distribution of ΔK. The proportion of each individual’s genotype in each cluster or breed (q) and the average membership proportions in each cluster (Q) were calculated.

## Supplementary information

Supplemtary figures and legends

Table S1

Table S2

Table S3
