# Author Correction: The genetic ancestry of American Creole cattle inferred from uniparental and autosomal genetic markers

**DOI:** 10.1038/s41598-020-73066-4

**Published:** 2020-10-06

**Authors:** Catarina Ginja, Luis Telo Gama, Oscar Cortés, Inmaculada Martin Burriel, Jose Luis Vega-Pla, Cecilia Penedo, Phil Sponenberg, Javier Cañón, Arianne Sanz, Andrea Alves do Egito, Luz Angela Alvarez, Guillermo Giovambattista, Saif Agha, Andrés Rogberg-Muñoz, Maria Aparecida Cassiano Lara, BioBovis Consortium, Sónia Afonso, Lenin Aguirre, Eileen Armstrong, Maria Esperanza Camacho Vallejo, Amado Canales, Bernardo Cassamá, Gloria Contreras, J. M. Moras Cordeiro, Susana Dunner, Ahmed Elbeltagy, Maria Clorinda Soares Fioravanti, Mayra Gómez Carpio, Mariano Gómez, Antonio Hernández, Darwin Hernandez, Raquel Soares Juliano, Vincenzo Landi, Ribamar Marques, Rubén D. Martínez, O. Roberto Martínez, Lilia Melucci, Baldomero Molina Flores, Fernando Mújica, Pere-Miquel Parés i Casanova, Jorge Quiroz, Clementina Rodellar, Gerald Tjon, Tumininu Adebambo, Odalys Ufo, Julio César Vargas, Axel Villalobos, Pilar Zaragoza, Juan Vicente Delgado, Amparo Martinez

**Affiliations:** 1 CIBIO/InBIO, Centro de Investigação em Biodiversidade e Recursos Genéticos, Universidade dDo Porto, Porto, Portugal; 2 CIISA.Faculdade de Medicina Veterinaria, Universidade de Lisboa, Lisbon, Portugal; 3 Departamento de Producción Animal, Facultad de Veterinaria, Universidad Complutense de Madrid, Madrid, Spain; 4 Laboratorio de Genética Bioquímica, Facultad de Veterinaria, Universidad de Zaragoza, Zaragoza, Spain; 5 Laboratorio de Investigación Aplicada, Servicio de Cría Caballar de Las Fuerzas Armadas, Córdoba, Spain; 6 Veterinary Genetics Laboratory, University of California, Davis, CA USA; 7 Virginia-Maryland Regional College of Veterinary Medicine, Virginia Tech, Blacksburg, USA; 8 Embrapa Gado de Corte, Campo Grande, Brazil; 9 Universidad Nacional de Colombia, Sede Palmira, Colombia; 10 Facultad de Ciencias Veterinarias, Universidad Nacional de La Plata, La Plata, Argentina; 11 Animal Production Department, Faculty of Agriculture, Ain Shams University, Cairo, Egypt; 12 CONICET, Buenos Aires, Argentina; 13 Centro de Genética e Reprodução, Instituto de Zootecnia, Nova Odessa-SP, Brazil; 14 Departamento de Genética, Facultad de Veterinaria, Universidad de Córdoba, Córdoba, Spain; 15 Animal Beeding Consulting S.L. Universidad de Córdoba, Córdoba, Spain; 16 Faculdade de Veterinária, Universidade Eduardo Mondlane, Maputo, Mozambique; 17 Universidad Nacional de Loja, Loja, Ecuador; 18 Departamento de Genética y Mejora Animal, Facultad de Veterinaria-UdelaR, Montevideo, Uruguay; 19 IFAPA Centro Alameda del Obispo, Córdoba, Spain; 20 Direçao Geral da Pecuária, Bissau, Guinea-Bissau; 21 Instituto Nacional de Investigaciones Agrícolas (INIA)-Venezuela, Maracay, Venezuela; 22 Faculdade de Medicina Veterinária, Universidade José Eduardo dos Santos, Huambo, Angola; 23 Department of Animal Biotechnology, Animal Production Research Institute, Ministry of Agriculture, Cairo, Egypt; 24 Universidade Federal de Goiás, Goiânia, Goiás Brazil; 25 Servicio de Ganadería, Diputación Foral de Bizkaia, Bizkaia, Spain; 26 Universidad Veracruzana, Veracruz, Mexico; 27 Embrapa Pantanal, Corumbá, MS Brazil; 28 EMBRAPA Amazônia Oriental, Belém, Pará Brazil; 29 Facultad de Ciencias Agrarias, Universidad Nacional de Lomas de Zamora, Zamora, Argentina; 30 Universidad Nacional de Asunción, Asunción, Paraguay; 31 Facultad de Ciencias Agrarias, Universidad Nacional de Mar del Plata, Balcarce, Argentina; 32 Facultad de Ciencias Agrarias, Universidad Austral de Chile, Santiago, Chile; 33 Universitat de Lleida, Lleida, Spain; 34 Instituto Nacional de Investigaciones Forestales, Agrícolas y Pecuarias, Mexico; 35 Ministry of Agriculture, Animal Husbandry and Fisheries, Paramaribo, Suriname; 36 University of Agriculture Abeokuta, Abeokuta, Nigeria; 37 Centro Nacional de Sanidad Agropecuaria, Blacksburg, Cuba; 38 Universidad Estatal Amazónica, Puyo, Pastaza Ecuador; 39 Instituto de Investigación Agropecuaria, Estación Experimental El Ejido, Los Santos, Panama

Correction to:* Scientific Reports* 10.1038/s41598-019-47636-0, published online 07 August 2019

This Article contains errors. The Acknowledgements section in this Article is incomplete, the funding source LISBOA-01-0145-FEDER-016647 is omitted,

“This work was supported by Animal Breeding Consulting S.L., Córdoba, Spain. This work was partially funded by the Veterinary Genetics Laboratory, University of California, Davis, VELOGEN S.L., Madrid, Spain and by Grupo de Referencia A19-17R LAGENBIO from Gobierno de Aragon/Fondo Social Europeo. C.G. was supported by Fundação Nacional para a Ciência e a Tecnologia (FCT), Portugal, Investigador FCT Grant IF/00,866/2014, and Project grant PTDC/CVTLIV/2827/2014 co-funded by COMPETE 2020 POCI-01-0145-FEDER-016647. The authors thank the collaboration of breeders, breed associations and “Red Iberoamericana Sobre la Conservacion de la Biodiversidad de Animales Domesticos Locales para el Desarollo Rural Sostenible (Red CONBIAND)” for the sharing of biological samples. Members of the CYTED XII-H and CONBIAND networks are thanked for valuable cooperation over the years. Authors thank Juan Antonio Pereira (FCV-UAGRM, Bolivia) and Olivier Hanotte for their support with sampling Criollo Yacumeño and Eastern Shorthorn Zebu respectively.”

should read:

“This work was supported by Animal Breeding Consulting S.L., Córdoba, Spain. This work was partially funded by the Veterinary Genetics Laboratory, University of California, Davis, VELOGEN S.L., Madrid, Spain and by Grupo de Referencia A19-17R LAGENBIO from Gobierno de Aragon/Fondo Social Europeo. C.G. was supported by Fundação Nacional para a Ciência e a Tecnologia (FCT), Portugal, Investigador FCT Grant IF/00,866/2014, Project grant PTDC/CVTLIV/2827/2014 co-funded by COMPETE 2020 POCI-01-0145-FEDER-016647 and LISBOA-01-0145-FEDER-016647. The authors thank the collaboration of breeders, breed associations and “Red Iberoamericana Sobre la Conservacion de la Biodiversidad de Animales Domesticos Locales para el Desarollo Rural Sostenible (Red CONBIAND)” for the sharing of biological samples. Members of the CYTED XII-H and CONBIAND networks are thanked for valuable cooperation over the years. Authors thank Juan Antonio Pereira (FCV-UAGRM, Bolivia) and Olivier Hanotte for their support with sampling Criollo Yacumeño and Eastern Shorthorn Zebu, respectively.”

In addition, a Data Availability section is not included in the article – it should appear as below:

“Data availability

STR data used in our analysis is available in the Dryad repository: https://doi.org/10.5061/dryad.5dv41ns43”.

