# Supplementary material for: The genetic ancestry of American Creole cattle inferred from uniparental and autosomal genetic markers
Source: Sci Rep. 2019 Aug 7;9:11486. doi: 10.1038/s41598-019-47636-0 (PMC6685949; doi:10.1038/s41598-019-47636-0)
Supplement: Supplementary file 1 — Supplemtary figures and legends [file 41598_2019_47636_MOESM1_ESM.pdf]

The genetic ancestry of American Creole cattle inferred from uniparental and autosomal genetic markers

Catarina Ginja[1§], Luis Telo Gama[2§], Oscar Cortés[3]*, Inmaculada Martín Burriel[4], José Luís Vega-Pla[5], Cecilia Penedo[6], Phil Sponenberg[7], Javier Cañón[3], Arianne Sanz[4], Andrea Alves do Egito[8], Luz Angela Alvarez[9], Guillermo Giovambattista[10], Saif Agha[11], Andrés Rogberg -Muñoz[12], Maria Aparecida Cassiano Lara[13], BioBovis Consortium[14], Juan Vicente Delgado[14+] and Amparo Martinez[14,15+]

[1]CIBIO/InBIO, Centro de Investigação em Biodiversidade e Recursos Genéticos, Universidade do Porto, Portugal.
[2]CIISA.Faculdade de Medicina Veterinaria, Universidade de Lisboa, Lisbon, Portugal.
[3]Departamento de Producción Animal, Facultad de Veterinaria, Universidad Complutense de Madrid, Spain.
[4]Laboratorio de Genética Bioquímica, Facultad de Veterinaria, Universidad de Zaragoza, Spain.
[5]Laboratorio de Investigación Aplicada, Servicio de Cría Caballar de las Fuerzas Armadas, Córdoba. Spain.
[6]Veterinary Genetics Laboratory, University of California, Davis, California, USA.
[7]Virginia-Maryland Regional College of Veterinary Medicine. Virginia Tech., Virginia, USA.
[8]Embrapa Gado de Corte, Campo Grande, Brazil.
[9]Universidad Nacional de Colombia, Sede Palmira, Colombia.
[10]Facultad de Ciencias Veterinarias, Universidad Nacional de La Plata, Argentina.
[11]Animal Production Department, Faculty of Agriculture, Ain Shams University, Cairo, Egypt.
[12]CONICET, Buenos Aires, Argentina.
[13]Instituto de Zootecnia, Centro de Genética e Reprodução, Nova Odessa-SP, Brasil.
[14]Departamento de Genética, Facultad de Veterinaria, Universidad de Córdoba, Córdoba, Spain.
[15]Animal Beeding Consulting S.L. Universidad de Córdoba, Córdoba, Spain.
* Corresponding author: ocortes@vet.ucm.es
[§] These authors contributed equally to this work
[+] These authors contributed equally to this work

Supp_Fig1_STR_TREE_Da_breeds.pdf: Neighbour-joining tree representation of Nei's DA genetic distances between 109 breeds based on microsatellite data and showing bootstrap values. Bootstrap values were obtained with 1,000 replicates over loci (only bootstrap values >30% are shown). Breed numerical codes are as defined in Table 1.

Supp_Fig2_STR_TREE_Da_groups.pdf: Neighbour-joining tree representation of Nei's DA genetic distances between geographic breed groups (cattle breeds were grouped according to their origin) based on autosomal microsatellite data. Bootstrap values were obtained with 1,000 replicates over loci. Breed groups are as defined in Table 1.

Supp_Fig3_distruct_summary.pdf: Contributions of ancestral populations to the 109 breeds analyzed and graphically represented with CLUMPAK (http://clumpak.tau.ac.il/), assuming values of K from K=2 a K=40. The coloured segment shows the breed's estimated membership proportions in a given cluster. Breed numerical codes are as defined in Table 1.

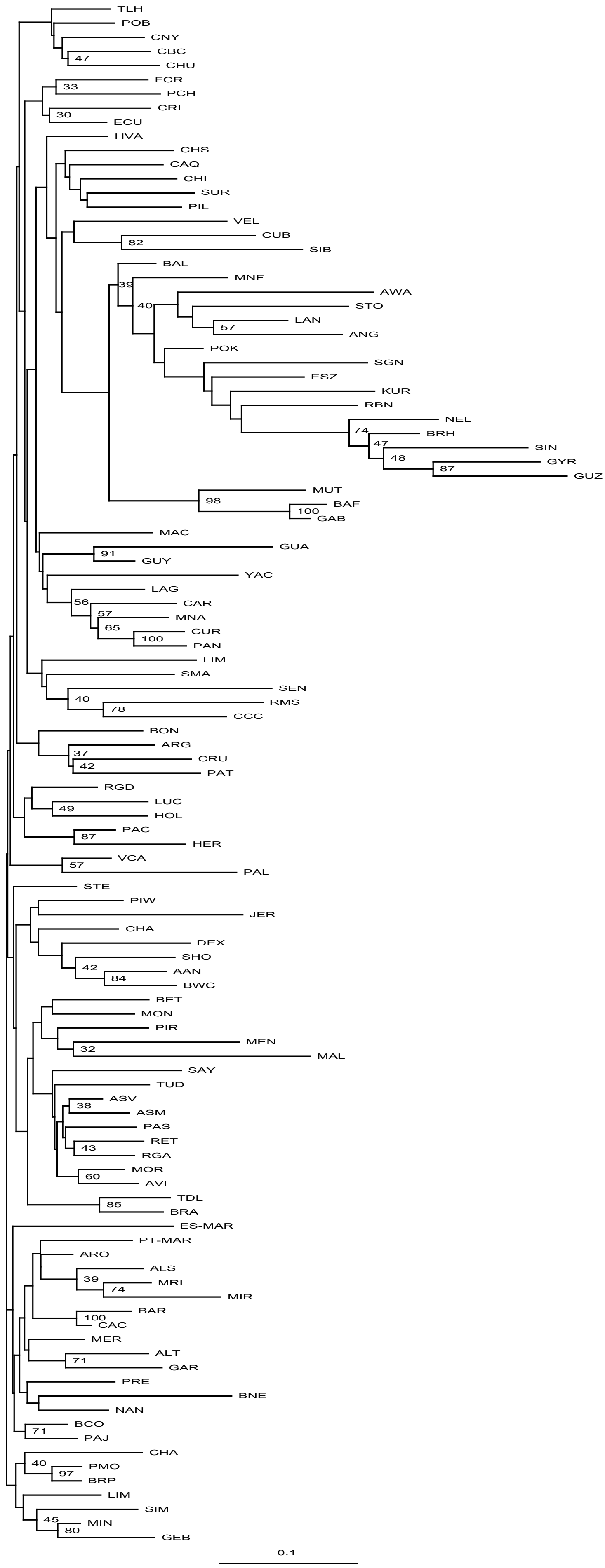

Supp_Fig1_STR_TREE_Da_breeds.pdf: Neighbour-joining tree representation of Nei's DA genetic distances between 109 breeds based on microsatellite data and showing bootstrap values. Bootstrap values were obtained with 1,000 replicates over loci (only bootstrap values >30% are shown). Breed numerical codes are as defined in Table 1.

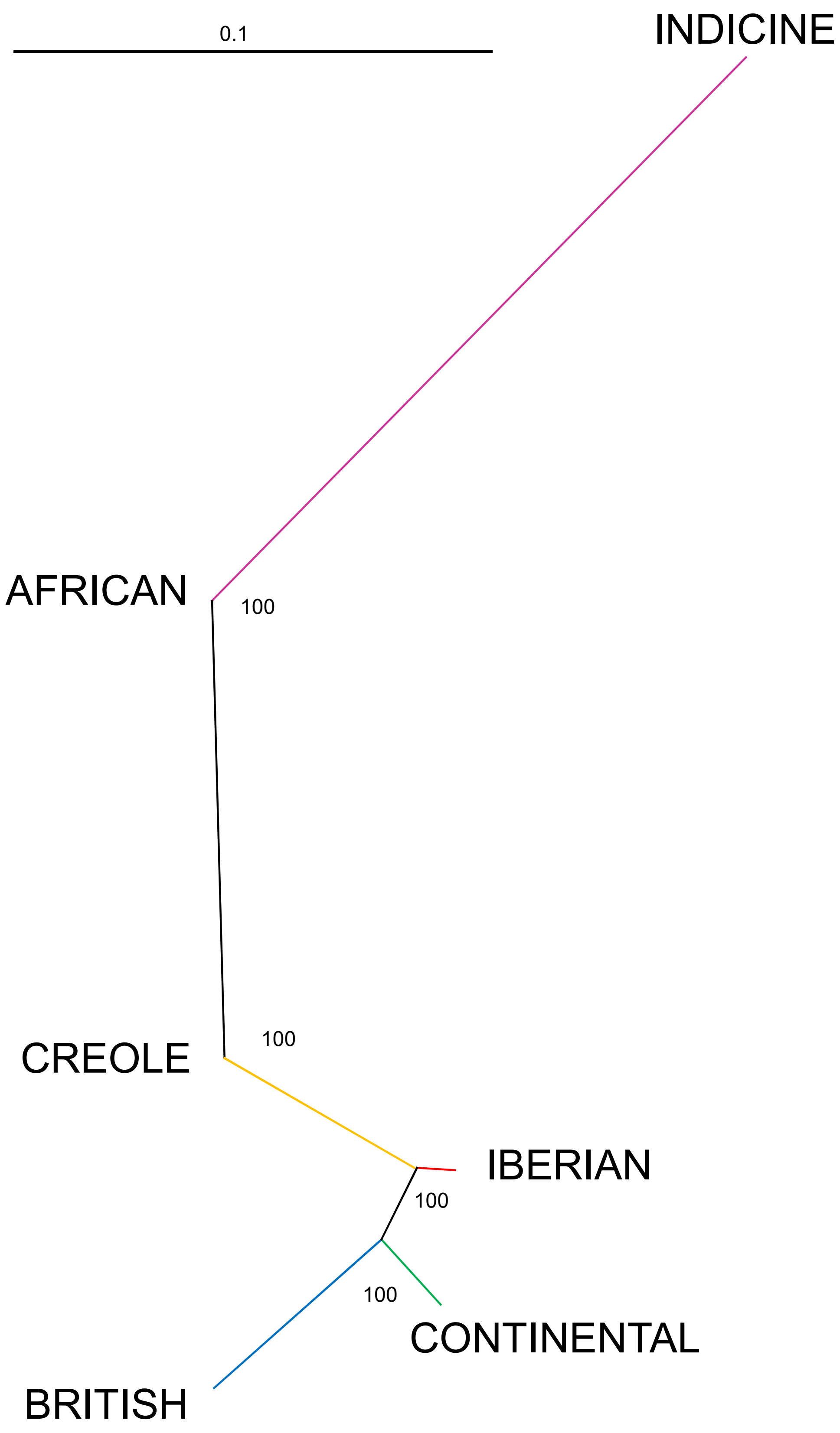

Supp_Fig2_STR_TREE_Da_groups.pdf: Neighbour-joining tree representation of Nei's DA genetic distances between geographic breed groups (cattle breeds were grouped according to their origin) based on autosomal microsatellite data. Bootstrap values were obtained with 1,000 replicates over loci. Breed groups are as defined in Table 1.

K=2 results-k2-r0-nodo91-f

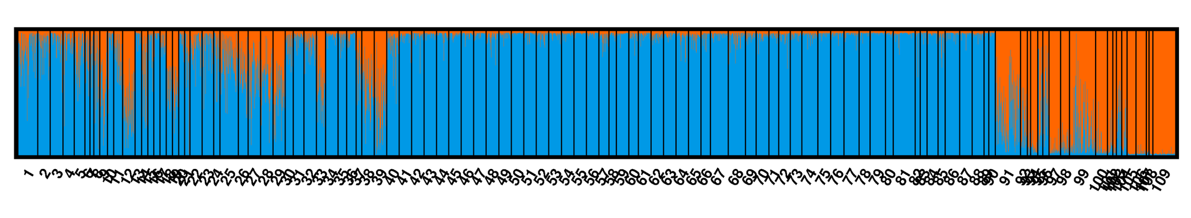

K=3 results-k3-r0-chuck1-f

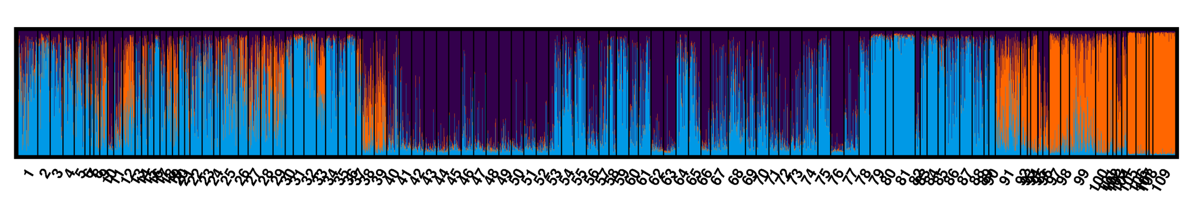

K=4 results-k4-r0-bigfoot-f

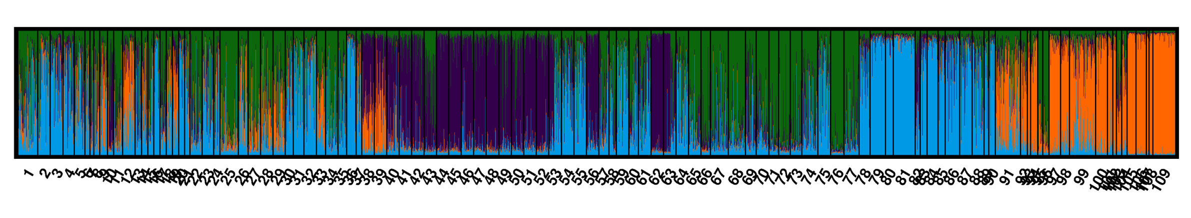

K=5 results-k5-r0-nova21-f

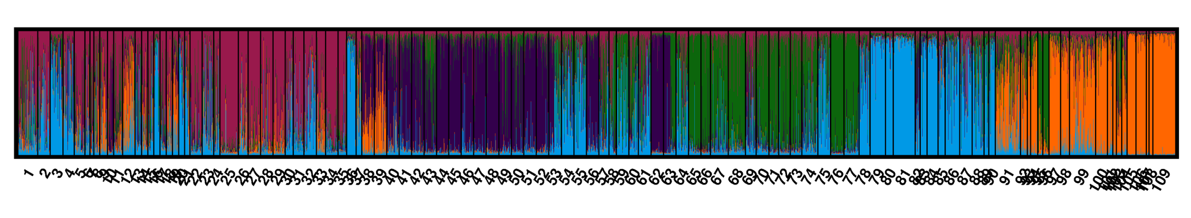

K=6 results-k6-r0-chuck4-f

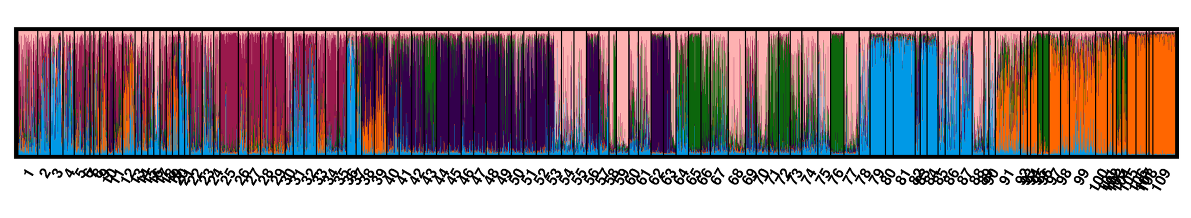

K=7 results-k7-r0-nova9-f

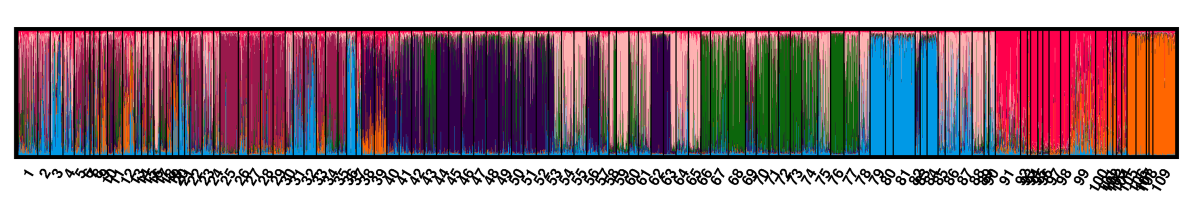

K=8 results-k8-r0-chuck1-f

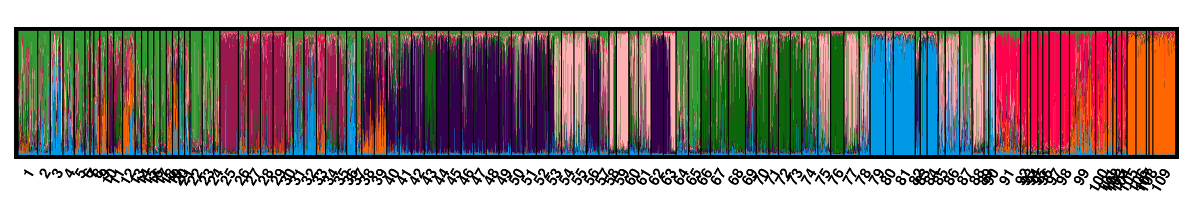

K=9 results-k9-r0-nova13-f

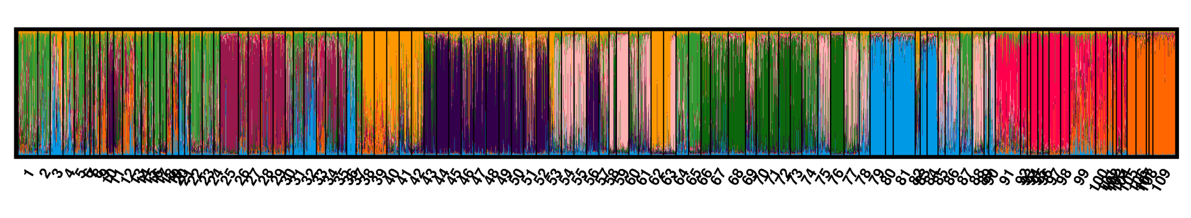

K=10 results-k10-r0-nodo135-f

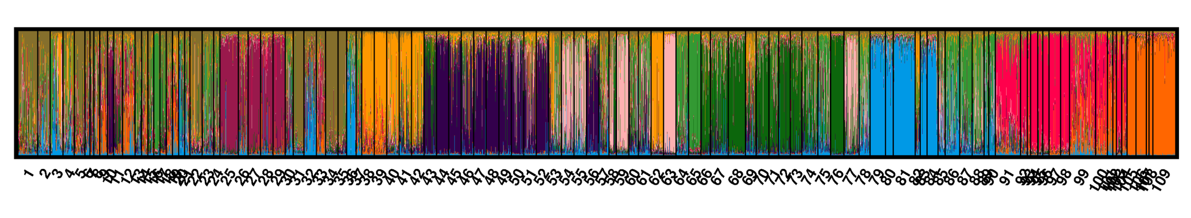

K=11 results-k11-r0-nodo91-f

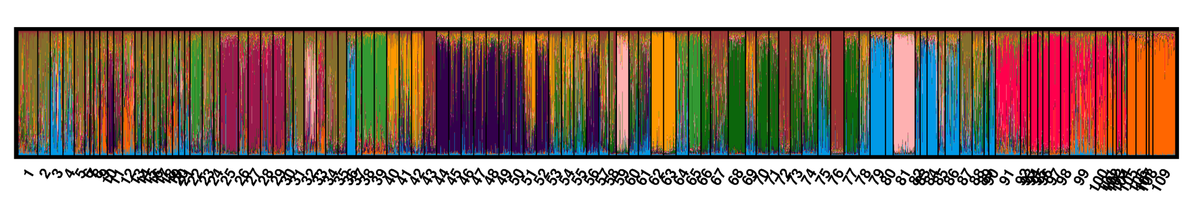

K=12 results-k12-r0-euler-f

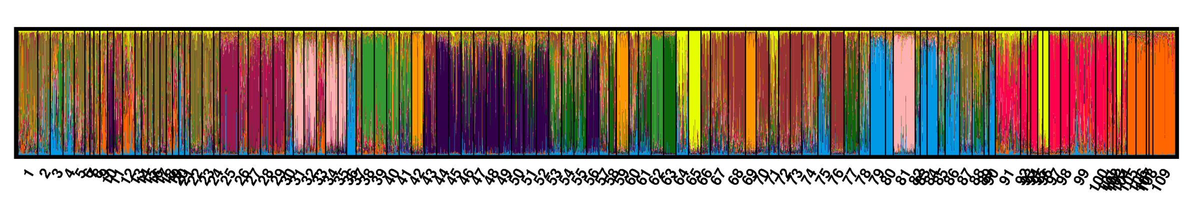

K=13 results-k13-r0-chuck2-f

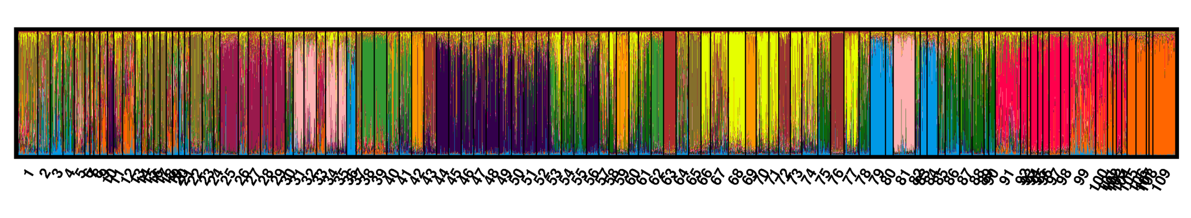

K=14 results-k14-r0-chuck1-f

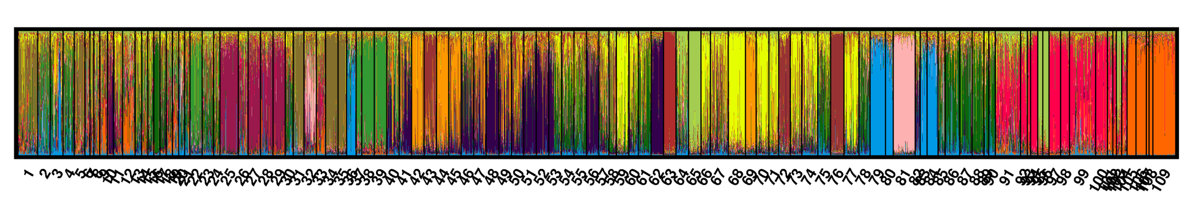

K=15 results-k15-r0-chuck4-f

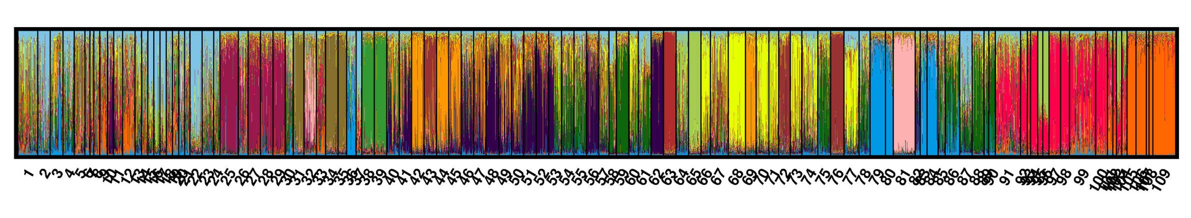

K=16 results-k16-r1-nova21-f

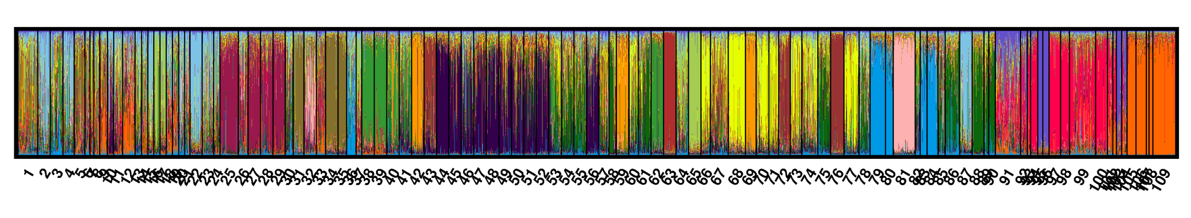

K=17 results-k17-r0-nova20-f

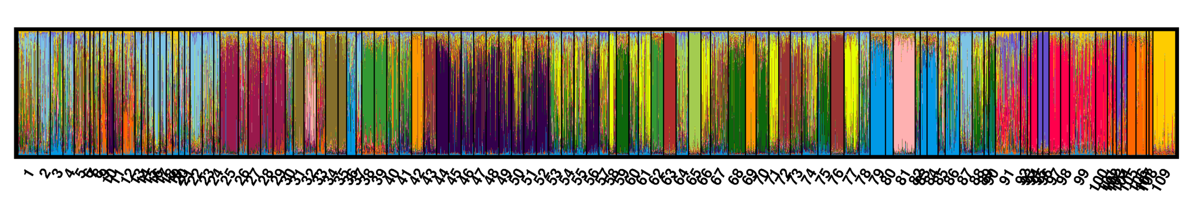

K=18 results-k18-r2-nodo82-f

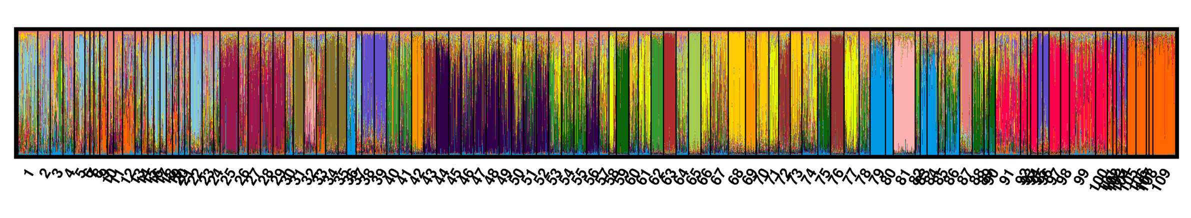

K=19 results-k19-r1-nodo123-f

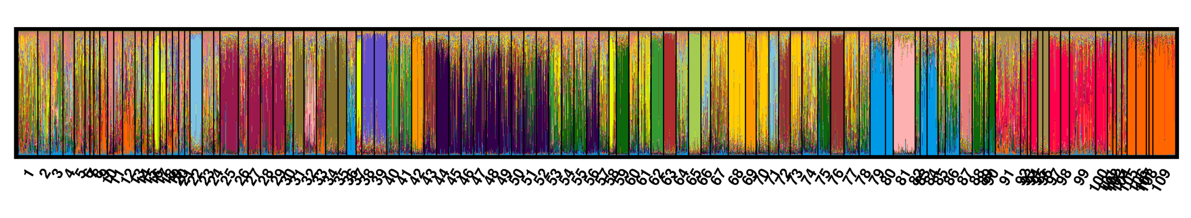

K=20 results-k20-r0-nova19-f

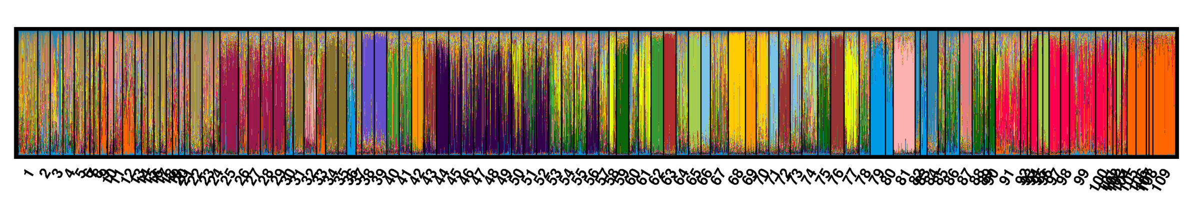

K=21 results-k21-r0-nodo135-f

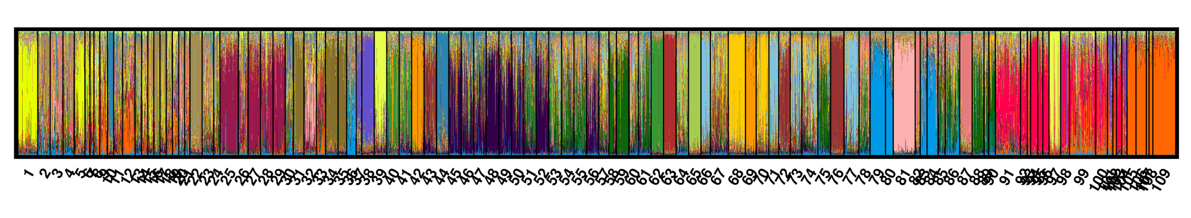

K=22 results-k22-r0-gazpacho-f

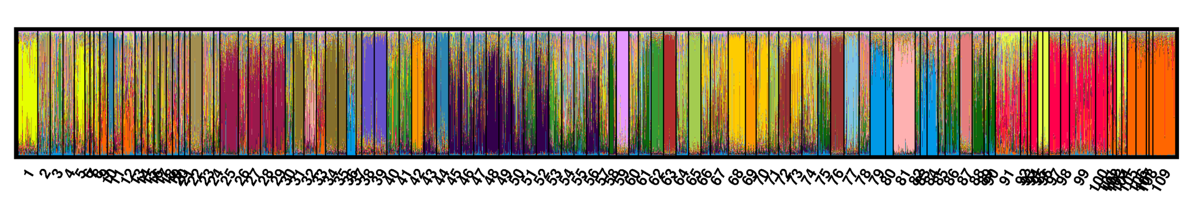

K=23 results-k23-r1-nova18-f

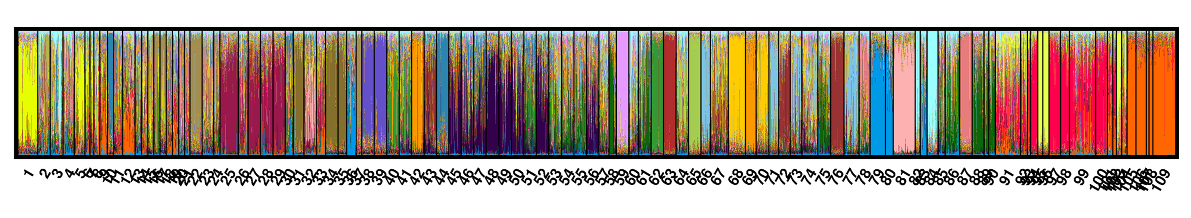

K=24 results-k24-r0-chuck3-f

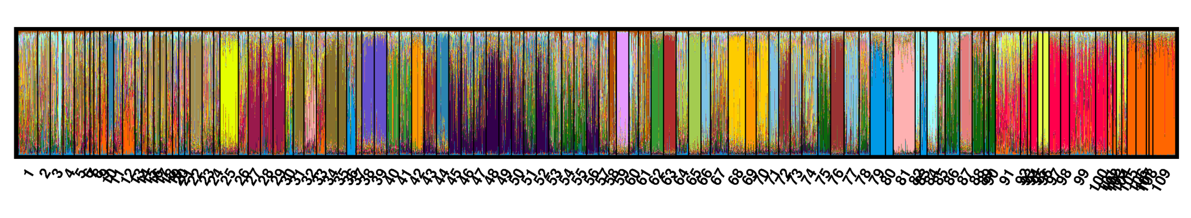

K=25 results-k25-r0-bigfoot-f

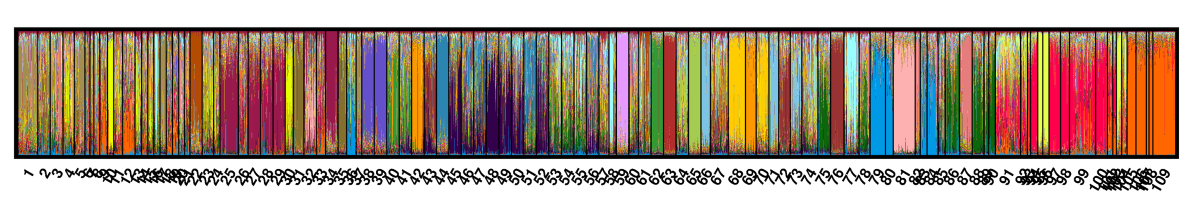

K=26 results-k26-r1-nodo108-f

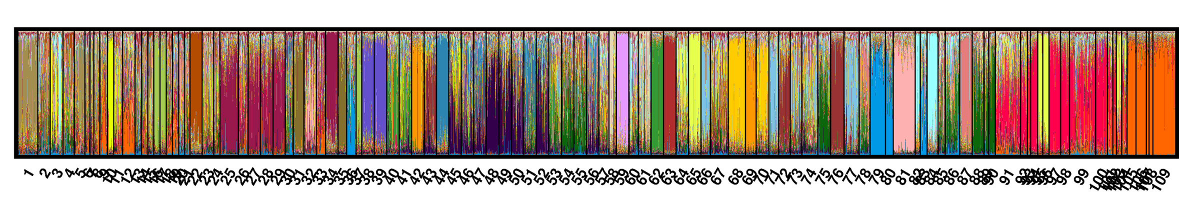

K=27 results-k27-r0-nova7-f

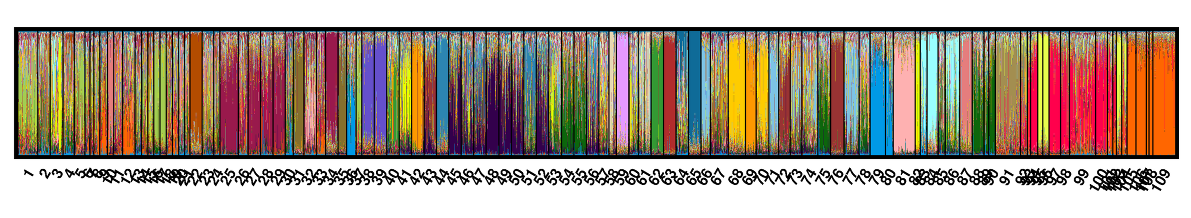

K=28 results-k28-r0-bigfoot-f

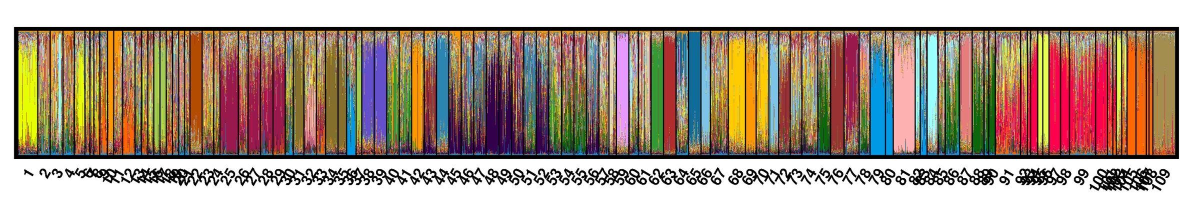

K=29 results-k29-r0-nova7-f

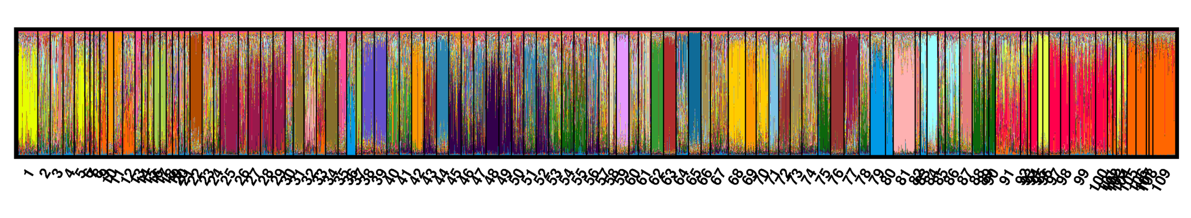

K=30 results-k30-r0-nova3-f

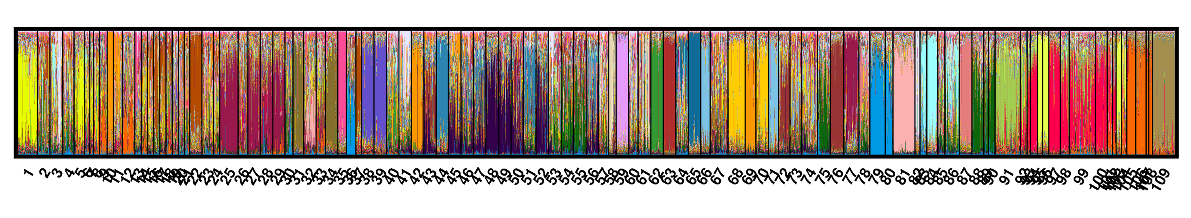

K=31 results-k31-r0-chuck1-f

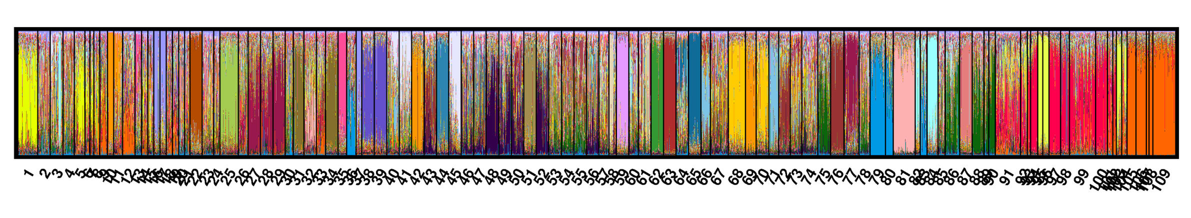

K=32 results-k32-r2-nova2-f

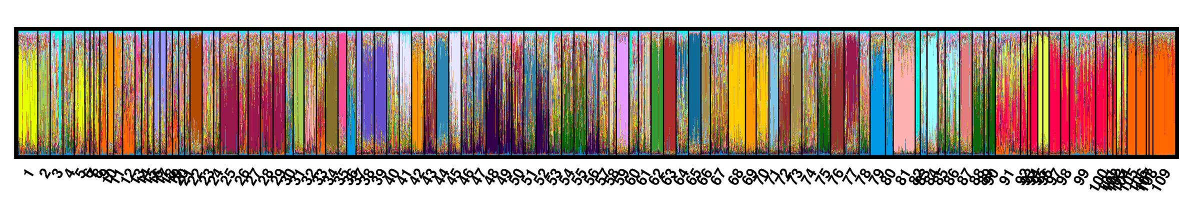

K=33 results-k33-r0-nova11-f

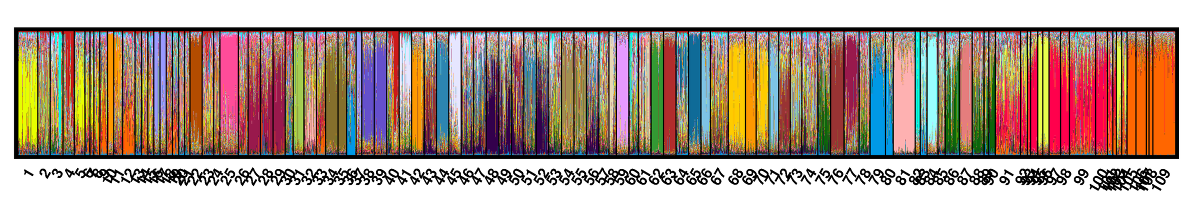

K=34 results-k34-r0-nodo92-f

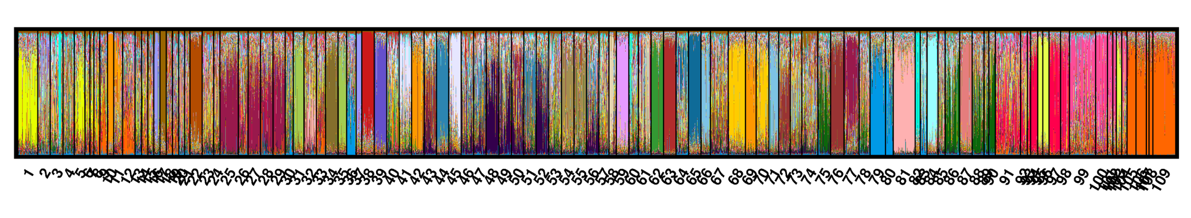

K=35 results-k35-r1-nova18-f

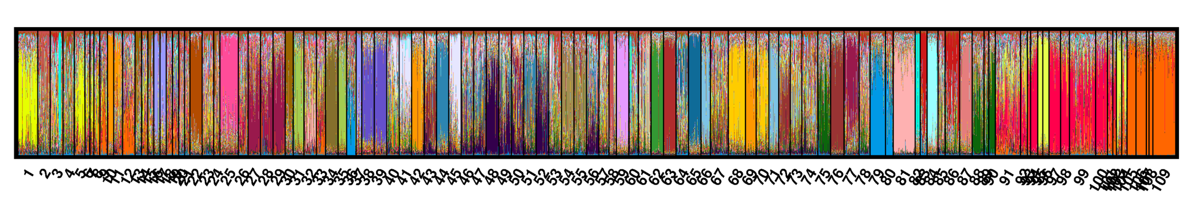

K=36 results-k36-r0-bigfoot-f

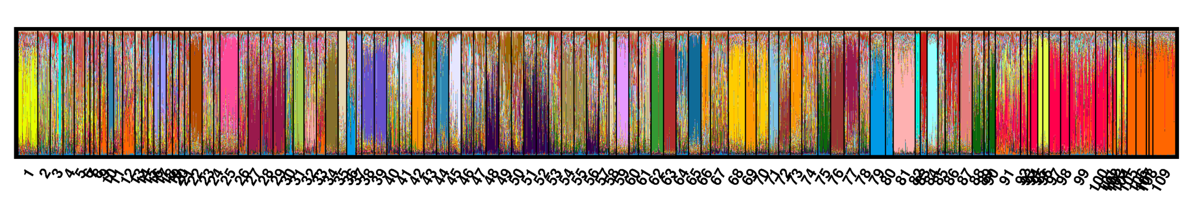

K=37 results-k37-r0-bigfoot-f

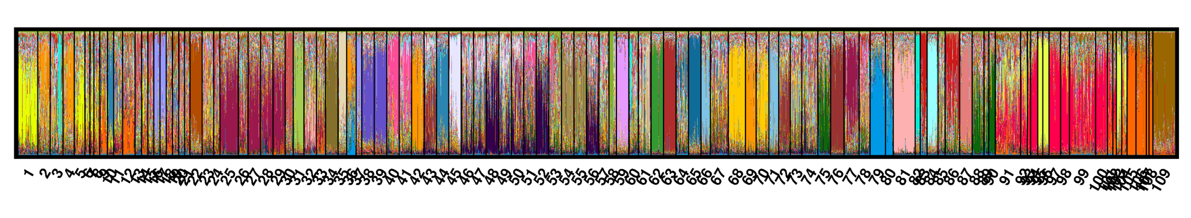

K=38 results-k38-r0-nodo123-f

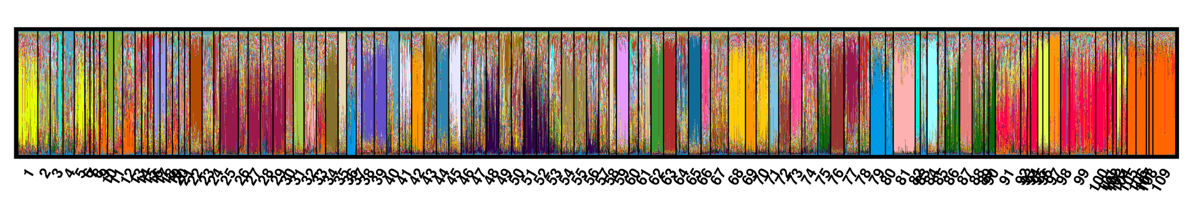

K=39 results-k39-r4-nova31-f

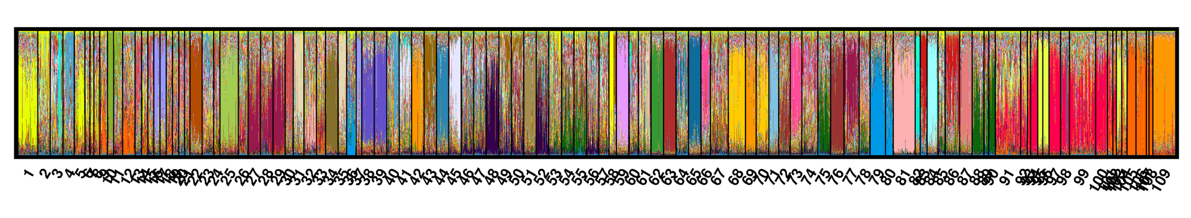

K=40 results-k40-r0-bigfoot-f

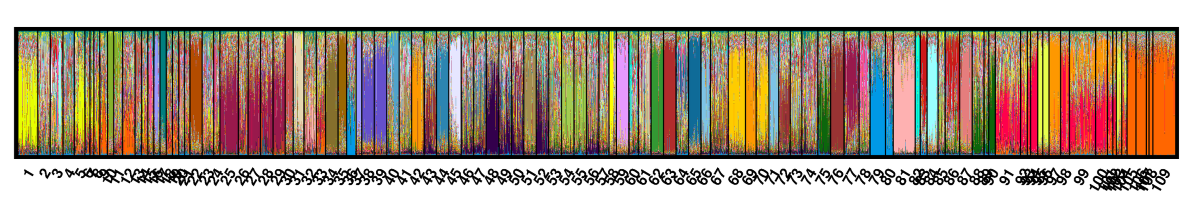

Supp_Fig3_distruct_summary.pdf: Contributions of ancestral populations to the 109 breeds analyzed and graphically represented with CLUMPAK (http://clumpak.tau.ac.il/), assuming values of K from K=2 a K=40. The coloured segment shows the breed's estimated membership proportions in a given cluster. Breed numerical codes are as defined in Table 1.
